# Challenges in the practical application of the Vienna test system for assessing cognitive functions in the general, athletic and clinical populations: a global scoping review of experimental and observational studies

**DOI:** 10.3389/fspor.2026.1716584

**Published:** 2026-02-23

**Authors:** János Négyesi, Péter Kovács, András Attila Horváth

**Affiliations:** 1 Department of Kinesiology, Hungarian University of Sports Science, Budapest, Hungary; 2 Neurocognitive Research Center, Nyírő Gyula National Institute of Psychiatry, and Addictology, Budapest, Hungary; 3 CRU Hungary Kft., Budapest, Hungary; 4 Department of Anatomy Histology and Embryology, Semmelweis University, Budapest, Hungary; 5 Faculty of Medicine, Universidade de Lisboa, Lisbon, Portugal

**Keywords:** cognitive skills, patients, sport, traffic, VTS

## Abstract

The Vienna Test System (VTS) is a widely used computerized tools for assessing psychology-related constructs in different populations. Our objectives were to (1) identify the available evidence on the use of VTS, (2) examine how research is conducted using VTS and, therefore, (3) draw attention to the challenges in the practical application of the VTS for assessing cognitive functions in general, athletic and clinical populations. A literature search following the JBI and PRISMA guidelines for scoping reviews was conducted in September 2024 across four databases (PubMed, Web of Science, ELSEVIER Scopus, and EBSCOhost) to identify peer-reviewed articles reporting cognitive functions measured by at least one VTS cognitive test. Of the 79 identified articles, 24 used the VTS to assess cognitive function in the general population, while 41 and 14 studies recruited participants from the athletic and clinical populations, respectively. Our analysis revealed RT (36.7% of total articles, *n* = 29), DT (35%, *n* = 28), COG (34.2%, *n* = 27), PP (19%, *n* = 15) and LVT (16.5%, *n* = 13) as the TOP 5 most popular tests in the VTS. Nevertheless, only two studies (2.5%, 2/79) received a modified JBI quality score above 70%, while 9 studies (11.4%, 9/79) scored between 50% and 70%. In addition, only 36.7% of the articles were published in Q1 journals, while almost one quarter (24%) were published in journals that do not even appear in the Scimago Journal Ranking. Overall, the wide spectrum of cognitive tests in the VTS has the potential to assess cognitive functions; however, quality assessments revealed that, e.g., the relatively poor experimental designs, lack of a control group, and inconsistent use of VTS tests of many included studies make it difficult to draw clear conclusions about their validity, feasibility, and reliability, highlighting the need for higher-quality studies.

## 1 Introduction

The human brain is presumably the most complex organ of all biological systems that has a fundamental role in movement initiation and sensation, and is responsible for many cardinal functions, including emotions, language, thinking, and memory (1). Although a broad spectrum of literature aims to understand the human cognitive function that emerges from neuronal structure and dynamics, it is still not entirely understood (2). In psychology, questionnaires are often used to permit valuable insights into an individual's cognitive functions (3). However, questionnaires might not be the most feasible way to obtain a broad profile of a participant, as they are typically designed to probe a specific aspect of cognition. Therefore, supplementary objective alternative measures are needed to collect a more diverse range of information about cognitive functions. In the last two decades, computer-based assessments, i.e., Concussion Resolution Index (CRI), ImPACT, and the CogSport have become widely used in sports sciences to identify instances of sports-related concussion (4). These methods seem to overcome the limitations of traditional measurement methods.

The Vienna Test System (VTS) is a widely used computerized tool for assessing psychology-related constructs across the general, athletic, and clinical populations. This system, developed by Schuhfried GmbH (Moedling, Austria), includes tests of many different constructs (5). The manufacturer's website (https://www.schuhfried.com) describes this comprehensive software platform, featuring over 120 modular, multilingual tests for work, health, and education. These tests evaluate cognitive skills, including intelligence, memory, attention, reaction time, and spatial abilities, as well as personality traits, such as personality, attitudes, behavior, and interests, with popular examples like reaction tests (RT), planning (TOL-F), stress tolerance (DT), risk-taking (WRBTV), and core intelligence (INSBAT) available in batteries or individually for areas like aptitude, fitness to drive, and sports performance. The platform's main features include the VTS software for test administration, automatic scoring, and reporting, complemented by specialized hardware for psychomotor tasks. This setup enables tailored test batteries for clinical, industrial/organizational (I/O), and sports psychology contexts. VTS is widely used by researchers, coaches, trainers, and practitioners to conduct cognitive testing in the general population, including younger and older adults, athletes across different sports, and patients with various neurocognitive diseases. Therefore, VTS could potentially be used to diagnose and/or develop cognitive skills relevant to general cognitive or sports performance.

The cognitive tests in the VTS became popular not only for the cognitive assessment of the general population but also for athletes and patients. For example, driving-related cognitive abilities were previously assessed using VTS tests in healthy older adults (6, 7), in car-racing drivers (8), and in patients who had completed previous tapentadol prolonged-release trials for severe low back or osteoarthritis pain (9). Nevertheless, the limitations of these studies are worthy of mentioning, i.e., (1) the VTS provides only a measure of driving-related skills but is not predictive of the actual driving behavior, (2) a selection bias of participants mostly prevents the generalization of the results, and (3) some of the VTS tests (naming or setup) are misleading. In the athletic population, some studies used the VTS not only to assess cognitive skills but also to induce mental fatigue. In a previous study (10), university first-team soccer players performed a modified Stroop task and VTS test. The results revealed that mental fatigue decreased players' visual field, as measured via the Peripheral Perception Test (PP) of the VTS; however, the changes observed in the study may have been due to prolonged mental effort prior to the field task. In addition, although the VTS is also used to assess the cognitive skills of patients with, e.g., ADHD (11), depression (12), Parkinson's disease (13), or schizophrenia (14–17), discrepancies even in the correct naming of the VTS tests make it difficult to draw clear conclusions.

Altogether, the use of VTS is supported by encouraging scientific evidence [for reviews, see (18, 19)], however, paralleling the inconsistencies in study designs, and the heterogeneity of VTS tests used in previous studies, the results are also contradictory concerning how, if at all, VTS have the potential to assess or improve cognitive functions in various populations. Therefore, the aims of this scoping review were to (1) identify the available evidence on the use of VTS, (2) examine how research is conducted using VTS, and, therefore, (3) draw attention to the challenges in the practical application of the VTS for assessing cognitive functions in general, athletic and clinical populations. In contrast to previous reviews, which were limited in their focus and discussed the use of VTS in sport psychology research (18, 19), we conducted a global scoping review that also considers its use in general and clinical populations. The heterogeneity of VTS tests used in previous studies prevented us from performing a meta-analysis; however, the analysis methods used in the current review might be more reliable for accurately estimating the potential and limitations of VTS.

## 2 Methods

### 2.1 Design

Scoping reviews aim to explore and test emerging concepts in a particular research area (20). Unlike typical systematic reviews, scoping reviews address broader and less-defined issues, often where relevant studies are unclear (21). Here, we aimed to provide a comprehensive critical review of the practical application of the VTS for assessing cognitive functions in general, athletic, and clinical populations. Therefore, to review a wide spectrum of literature and evaluate the application of the Vienna Test System to assess cognitive function in different populations, the Joanna Briggs Institute (JBI) guidelines (22) and the Preferred Reporting Items for Systematic Reviews and Meta-Analyses Extension for Scoping Reviews (PRISMA-ScR) were followed (23).

### 2.2 Review question

The review question used the population, concept, and context (PCC) strategy. General, athletic, and clinical populations formed the population; whereas, VTS cognitive tests and effective indication of cognitive skills constituted the concept and context, respectively. Thus, the final review question was: will VTS cognitive tests effectively indicate (1) the changes in cognitive skills after an intervention and (2) the differences between certain group of people in the general, athletic and clinical populations?

### 2.3 Search strategy

A systematic literature search based on the PCC model was conducted across four databases (PubMed, Web of Science, ELSEVIER Scopus, and EBSCOhost) in September 2024 by the lead author using the default field search settings in each database. This strategy provided coverage of the health, technology-related, and psychosocial literature and aligning with the JBI guidelines for multidisciplinary and topic-specific databases (22).

A combination of keywords related to VTS, cognition, and cognitive function was used with the Boolean conjunctions AND, OR, and NOT. The detailed search strategy has been provided in the Supplementary Material. Only original full-text peer-reviewed papers written in English were classified as relevant, with no restrictions on year of publication, to ensure all relevant literature was identified for screening. Conference papers and case-studies were excluded, as were reviews, but their references were manually screened to ensure all appropriate citations were also considered for inclusion.

### 2.4 Study selection and inclusion criteria

The selection process started with the removal of duplicate studies obtained from the databases using the EndNote bibliographic manager. After removing records, the titles and abstracts were independently screened by two authors (JN and AAH) using the inclusion and exclusion criteria. No discrepancies appeared regarding study eligibility; therefore, analyzing inter-reviewer agreement was not necessary. Studies eligible for inclusion were those that measured cognitive functions using at least one VTS cognitive test. Both parallel and crossover randomized controlled trials (RCTs) and intervention studies written in English were classified as relevant. Studies were excluded from the review if they: (1) measured not cognitive but motor skills with VTS or (2) measured cognitive function via a different experimental protocol or method than VTS. Because the heterogeneity of VTS outcome variables prevented us from performing a meta-analysis, the lack of presenting means and SD was not considered as an exclusion criterion. Nevertheless, the study was excluded from the present scoping review due to inappropriate data reporting. Conference papers and study protocols were excluded, as were reviews, but their references were manually screened to ensure all appropriate citations were also considered for inclusion. Moreover, the study was excluded if the full text was not available, even upon request.

### 2.5 Data extraction

Data extraction was conducted in two phases. First, the lead author (JN) collected data from each study focusing on (1) general data (e.g., year of publication, sample population, and sample size); (2) participant characteristics (including age, height, and mass); (3) study objectives; (4) intervention characteristics; (5) VTS outcome measures (tests and package if relevant); and (6) key findings (Supplementary Tables S1–S3). After the initial data extraction, the information from all studies was reviewed by another author (AAH) to verify its accuracy. This method ensured data reliability, reducing the likelihood of errors or discrepancies. There were no discrepancies between the two authors.

### 2.6 Data synthesis strategy

**Figure 1:**
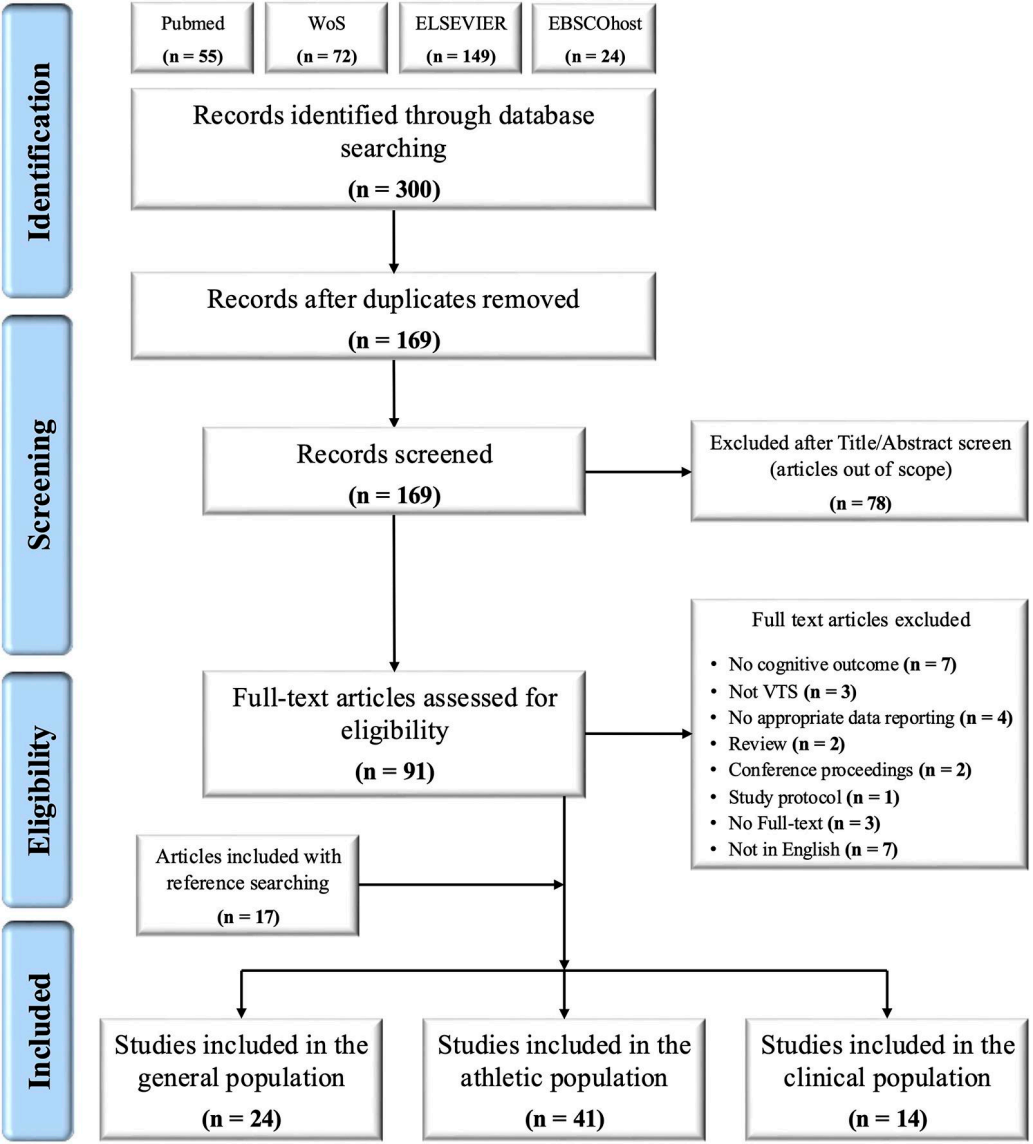
Process of study selection from initial identification to inclusion as per the preferred reporting items for systematic reviews and meta-analyses extension for scoping reviews (PRISMA-ScR).

The evidence from the included studies is summarized in tables, figures, and narrative form as per guidelines (22). The PRISMA flowchart represents the stratification of the selected studies (Figure 1). Supplementary Tables S1–S3 provide a summary of the general characteristics of the included studies in the general, athletic and clinical populations, respectively.

### 2.7 Quality assessment

Although the VTS is widely used across general, athletic, and clinical populations, the quality of published manuscripts makes it difficult to draw clear conclusions about its validity and feasibility. To assess the quality of the involved articles, a modified version (90) of the Joanna Briggs Institute (JBI) critical appraisal checklist (91) was used. The JBI tool was created after a thorough peer review and has received approval from the JBI Scientific Board. It outlines nine criteria for assessing a study's methodological quality and how well potential bias in its design, execution, and analysis has been managed. Results were reported as “yes”, “no”, or “unknown” for each criterion, with a summary expressed as the percentage of “yes” responses. In addition, the highest Scimago Journal Ranking of each paper in the year of publication was also considered. If the study was published in 2024, the previous year's SJR was used.

## 3 Results

### 3.1 Search results

An overview of the search process is displayed in Figure 1. Initial database searches identified 300 articles. After removing duplicates, 169 records remained. We screened out 78 records based on titles and abstracts. Of the remaining 91 records, 29 were excluded during the eligibility check based on the *a priori*-defined exclusion criteria. Seventeen additional studies (46, 51, 52, 55, 64–66, 68, 74, 75, 78–83, 92) were identified through reference list searches; therefore, a total of 79 articles were included in the scoping review. Twenty-four articles used the VTS for assessing cognitive function in the general population, while 41 and 14 studies recruited participants from the athletic and clinical populations, respectively.

### 3.2 General characteristics of the included articles

The total number of participants in the data set for the general population was 1,251 [*n* = 348 females and *n* = 847 males, one study (27) did not provide information on gender], with a mean age of 42 years (range between 18 and 92 years). One study solely enrolled female participants, eight enrolled only male participants, and 14 enrolled both females and males. Seven studies recruited students (26, 32, 34, 35, 37, 38, 40), eight studies recruited adults (24, 27–31, 44), six studies recruited older adults (7, 25, 33, 36, 39, 42), and three studies recruited both younger and older participants (6, 41, 43). From these studies, some assessed cognitive functions using the VTS in air traffic controllers (28), drivers (7, 25, 27), introverts and extroverts (26), active kava drinkers (24), maritime pilots (41), police officers (30), or train drivers (27). Five were analytical case-control studies (24, 26–28), three were analytical correlational studies (7, 30, 36), four studies were analytical longitudinal studies (25, 29, 39, 42), three were comparative studies (6, 34, 43), two were quasi-experimental studies (40, 41), four were parallel RCTs (31, 35, 38, 44), and three were cross-over RCTs (32, 33, 37). From these, 50% were intervention studies that e.g., ranged from waterpipe smoking (40) through balance training (42) to Korean red ginseng intake (44). The full list of interventions and the characteristics of each study are summarized in Supplementary Table S1.

Regarding the studies that measured the cognitive skills with the VTS in the athletic population, the total number of participants was 1,789 [*n* = 256 females and *n* = 1,316 males, seven studies (47, 52, 56, 76, 79, 80, 83) did not provide information on gender] with a mean age of 21.2 years (range between 10 and 64 years). Seven studies enrolled only female participants, twenty studies enrolled only male participants, and seven studies included both females and males. Five recruited students (10, 51, 67, 74, 83), twenty-three recruited adults (8, 46, 47, 52, 53, 56–65, 70, 75–77, 79–82), nine recruited younger participants (45, 48–50, 54, 68, 71, 73, 78), one recruited older participants (72), and three recruited both younger and older participants (55, 66, 69). The participants' field of sport in the studies included in the scoping review ranged from yoga-practicing older females (72) to professional athletes (e.g., racing drivers (8, 46), soccer players (49, 71), wrestlers and taekwondo competitors (52, 68), Polish amateur boxers (61), rhythmic gymnasts (78)). Six studies were analytical case-control studies (45, 46, 61, 64, 80, 82), seven studies were analytical correlational studies (48, 54, 65, 68, 71, 76, 79), seven studies were analytical longitudinal studies (50, 57, 63, 67, 70, 81, 83), eight studies were comparative studies (47, 49, 52, 55, 58, 60, 69, 78), one was a controlled case-study (66), one was a non-randomized controlled parallel study (74), one was a non-randomized cross-over study (73), two studies were non-randomized non-controlled (59, 62), six studies were parallel RCTs (8, 10, 53, 56, 72, 75), one study was cross-over RCTs (77), and one study was a reliability and validity study (51). From these, 17 were intervention studies that e.g., ranged from cognitive training program (10, 50, 53, 62, 70) through high-load training (64, 73, 81, 83) to sport-specific training (65, 77). The characteristics of each study that aimed to assess cognitive functions using the VTS in the athletic population are summarized in Supplementary Table S2.

Finally, 14 studies recruited a total of 1,707 participants from the clinical populations [*n* = 685 females and *n* = 942 males, two studies (12, 16) did not provide information on gender] with a mean age of 45.4 years (range between 18 and 69 years). Each study enrolled both females and males. Studies assessed cognitive functions using the VTS in patients with ADHD (11), chronic excessive daytime sleepiness (87), chronic obstructive pulmonary disease (84), depression (12), hypersomnolence (86), opioid dependency (89), pain (9, 88), Parkinson's disease (13), pneumococcal meningitis (85), and schizophrenia (14–17). Five studies were intervention studies that ranged from medication therapy (9, 13, 16) through pharmacotherapy and/or psychotherapy interventions (12) to sedentary behavior and physical activity (17). Except for one phase 3b trial (9), each study was an analytical case-control study. The characteristics of each study in the clinical population are summarized in Supplementary Table S3.

### 3.3 Popularity of tests for computerized psychological assessments in the VTS

**Figure 2:**
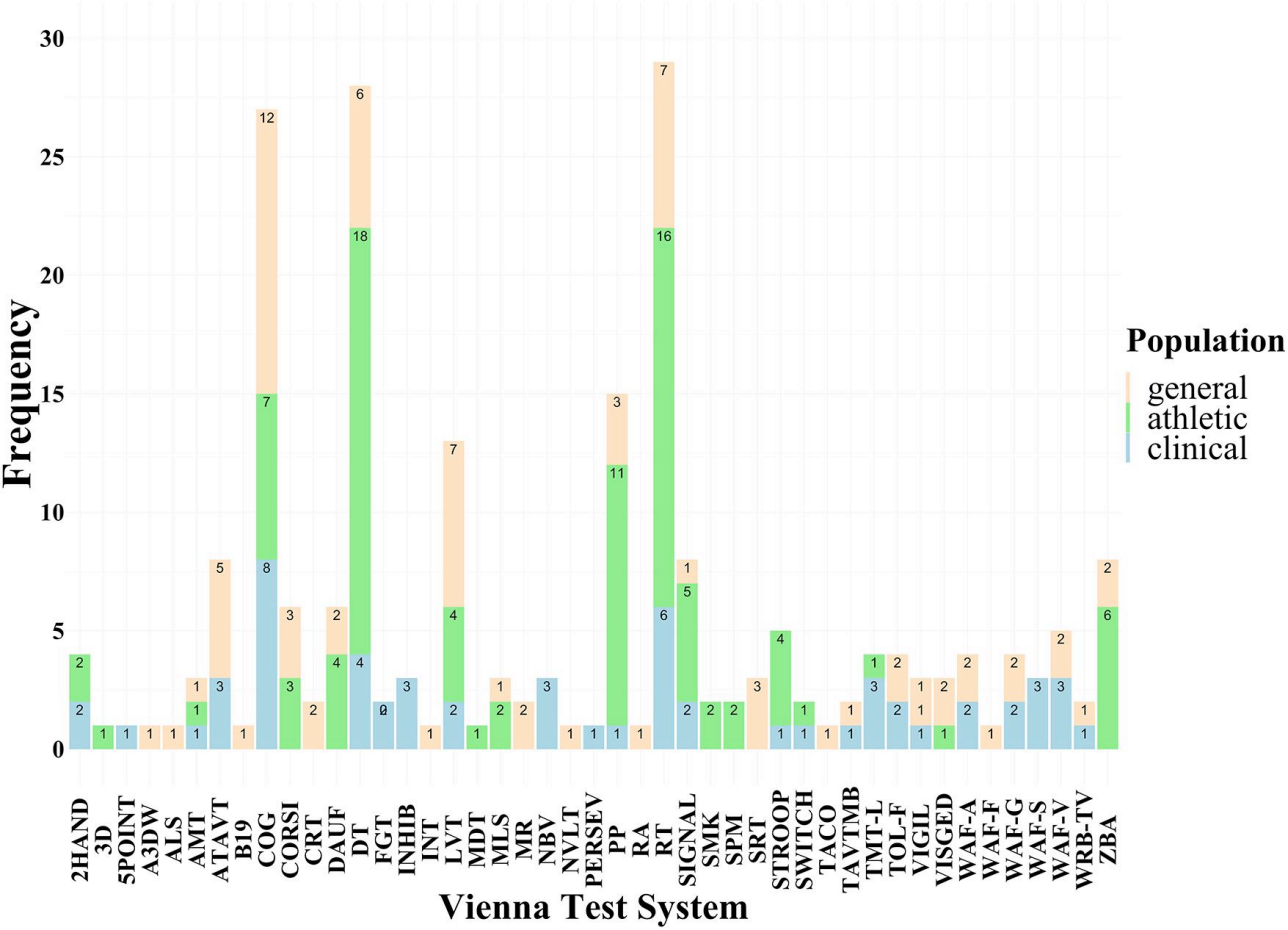
The list and frequency of each test in the general- (light brown), athletic (green), and clinical (blue) populations.

The wide spectrum of cognitive tests in the VTS has the potential to assess, e.g., sustained attention, reaction time, peripheral perception, stress reactivity, or time-movement anticipation. Our analysis revealed RT (36.7% of total articles, *n* = 29), DT (35%, *n* = 28), COG (34.2%, *n* = 27), PP (19%, *n* = 15), and LVT (16.5%, *n* = 13) as the TOP 5 most popular tests in the VTS. The list and frequency of each test in each population are shown in Figure 2.

### 3.4 Quality of articles

#### 3.4.1 According to the joanna briggs institute (JBI) critical appraisal tool

Two studies (2.5%, 2/79) received a modified JBI quality score above 70%, while 9 studies (11.4%, 9/79) scored between 50% and 70%. Most studies received an “unknown” rating for whether they conducted data analysis with complete coverage of the identified sample (100%, 79/79) and for reporting interobserver comparisons when multiple observers were involved in data collection (98.7%, 78/79). Only 17.7% (14/79) of studies performed a pre-statistical power analysis to estimate a minimum sample size for adequate precision of the prevalence estimate. Nearly all PPS scored a “yes” for sampling study participants in an appropriate way (96.2%, 76/79) and for reporting an appropriate measure of variability (94.9%, 75/79) (Table 1).

**Table 1:** Modified joanna briggs institute (JBI) critical appraisal checklist.

Study	Q1	Q2	Q3	Q4	Q5	Q6	Q7	Q8	Q9	Q10	Q11
General population											
Aporosa et al. (24)	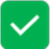	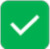	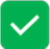	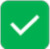	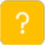	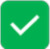	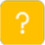	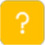	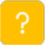	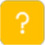	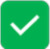
Balzarotti et al. (25)	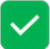	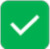	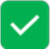	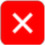	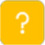	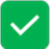	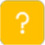	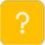	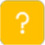	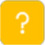	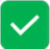
Bartolacci et al. (6)	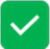	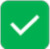	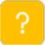	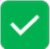	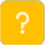	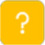	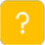	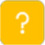	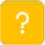	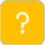	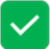
Deng & Wu (26)	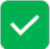	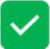	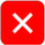	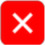	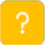	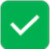	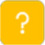	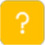	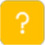	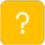	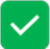
Grundler & Strasburger (7)	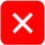	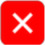	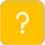	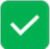	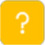	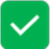	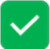	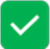	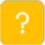	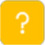	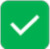
Hani Tabai et al. (27)	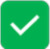	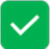	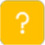	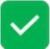	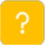	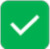	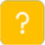	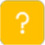	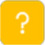	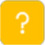	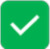
Hedayati et al. (28)	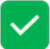	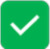	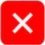	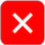	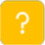	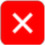	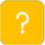	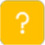	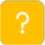	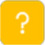	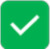
Jiménez-Pavón et al. (29)	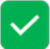	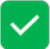	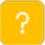	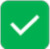	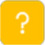	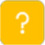	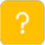	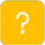	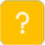	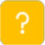	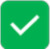
Johnsen et al. (30)	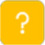	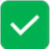	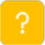	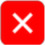	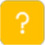	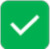	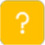	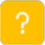	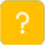	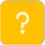	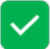
Laux & Corazza (31)	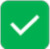	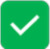	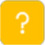	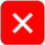	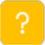	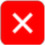	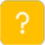	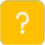	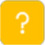	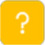	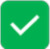
Loch et al. (32)	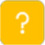	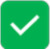	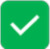	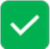	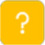	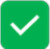	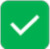	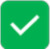	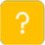	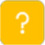	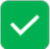
Malik et al. (33)	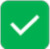	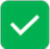	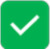	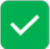	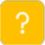	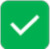	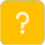	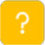	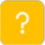	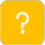	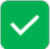
Mehri et al. (34)	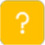	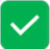	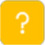	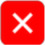	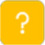	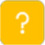	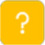	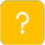	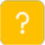	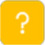	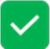
Mikicin (35)	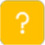	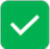	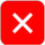	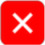	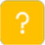	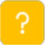	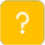	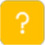	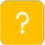	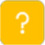	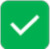
Mudrák & Slepicka (36)	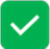	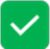	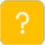	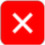	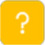	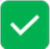	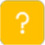	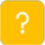	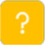	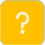	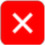
Müller et al. (37)	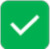	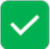	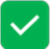	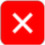	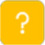	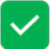	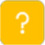	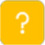	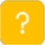	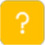	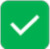
Niedzielska et al. (38)	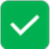	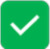	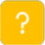	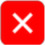	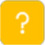	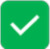	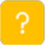	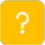	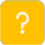	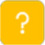	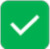
Roman-Liu & Mockałło (39)	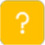	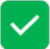	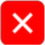	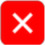	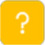	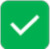	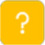	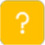	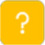	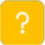	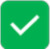
Saadat et al. (40)	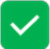	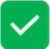	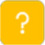	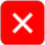	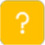	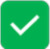	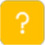	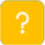	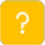	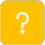	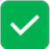
Seyfzadehdarabad et al. (41)	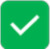	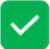	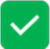	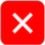	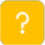	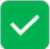	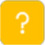	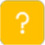	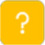	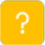	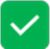
Tabai et al. (27)	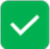	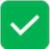	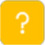	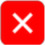	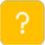	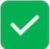	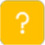	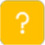	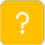	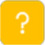	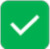
Taheri & Irandoust (42)	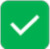	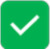	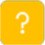	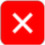	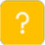	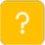	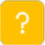	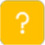	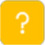	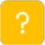	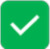
Tinella et al. (43)	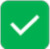	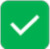	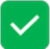	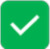	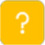	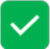	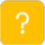	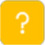	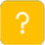	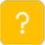	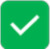
Yeo et al. (44)	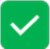	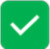	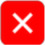	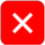	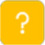	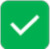	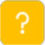	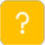	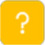	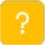	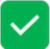
Athletic population											
Baláková et al. (45)	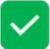	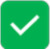	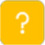	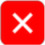	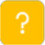	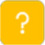	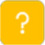	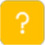	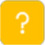	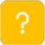	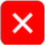
Baur et al. (46)	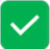	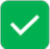	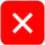	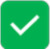	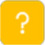	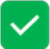	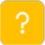	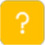	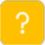	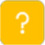	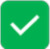
Blecharz et al. (47)	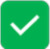	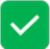	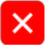	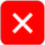	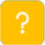	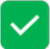	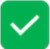	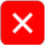	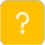	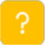	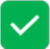
de Andrade et al. (48)	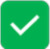	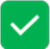	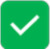	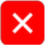	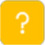	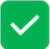	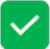	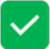	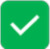	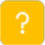	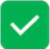
de Sousa Pinheiro et al. (49)	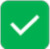	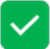	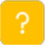	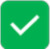	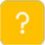	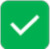	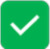	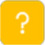	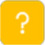	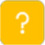	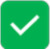
Fózer-Selmeci et al. (50)	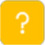	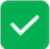	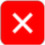	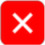	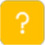	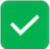	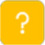	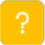	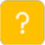	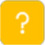	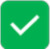
Gierczuk & Ljach (51)	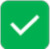	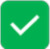	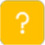	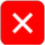	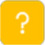	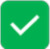	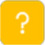	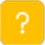	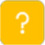	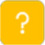	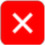
Gierczuk et al. (52)	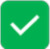	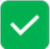	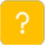	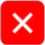	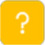	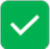	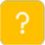	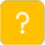	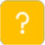	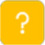	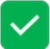
Horváth et al. (8)	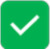	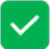	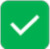	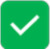	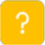	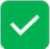	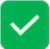	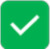	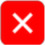	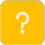	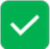
Horváth et al. (53)	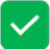	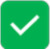	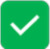	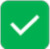	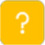	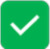	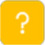	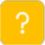	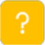	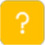	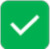
Huzarska et al. (54)	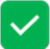	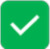	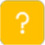	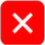	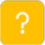	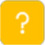	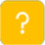	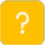	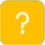	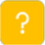	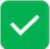
Johne et al. (55)	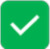	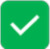	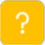	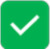	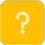	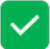	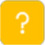	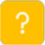	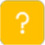	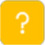	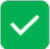
Kapur & Joshi (56)	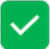	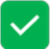	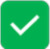	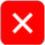	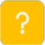	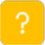	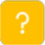	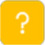	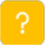	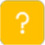	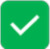
Khani et al. (57)	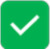	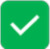	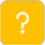	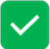	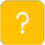	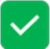	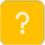	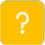	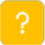	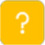	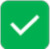
Kiss & Balogh (58)	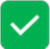	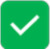	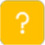	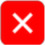	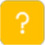	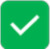	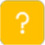	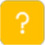	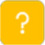	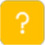	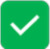
Kiss et al. (59)	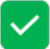	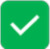	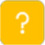	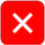	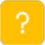	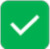	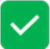	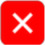	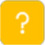	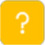	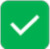
Kunrath et al. (10)	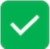	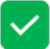	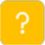	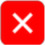	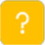	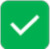	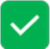	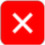	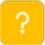	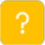	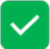
Kutlu et al. (60)	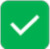	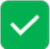	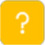	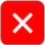	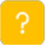	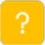	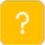	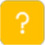	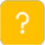	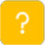	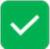
Lesiakowski et al. (61)	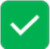	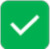	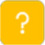	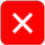	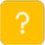	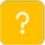	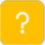	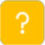	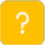	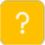	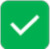
Liu et al. (62)	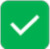	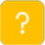	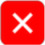	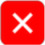	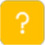	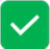	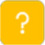	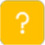	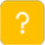	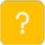	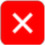
Mikicin & Szczypińska (63)	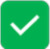	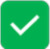	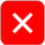	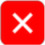	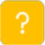	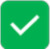	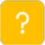	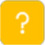	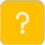	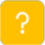	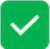
Nederhof et al. (64)	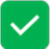	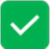	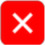	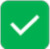	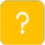	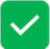	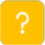	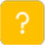	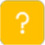	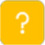	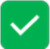
Nederhof et al. (65)	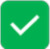	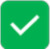	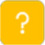	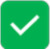	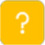	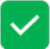	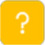	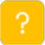	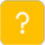	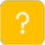	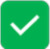
Nederhof et al. (66)	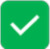	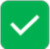	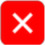	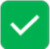	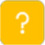	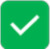	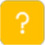	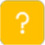	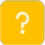	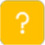	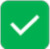
Pahan & Singh (67)	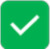	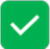	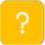	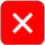	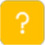	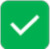	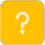	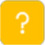	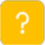	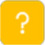	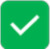
Sadowski et al. (68)	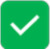	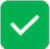	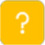	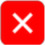	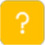	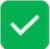	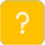	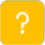	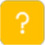	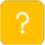	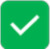
Schumacher et al. (69)	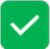	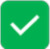	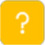	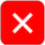	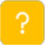	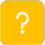	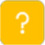	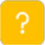	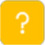	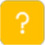	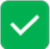
Szczypińska & Mikicin (70)	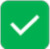	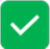	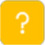	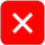	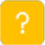	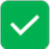	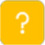	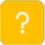	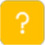	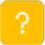	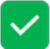
Szwarc et al. (71)	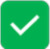	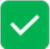	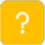	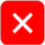	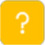	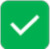	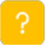	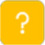	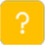	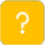	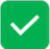
Taheri et al. (72)	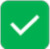	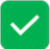	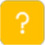	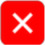	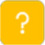	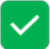	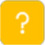	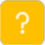	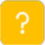	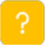	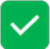
Teoldo et al. (73)	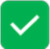	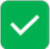	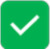	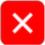	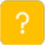	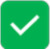	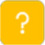	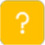	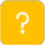	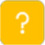	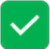
Tsorbatzoudis et al. (74)	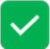	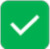	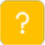	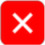	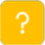	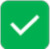	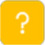	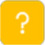	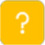	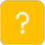	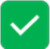
Tulppo et al. (75)	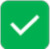	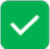	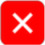	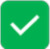	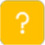	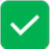	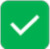	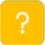	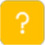	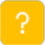	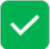
Wilczyńska (76)	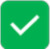	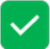	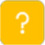	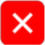	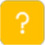	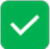	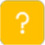	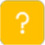	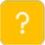	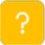	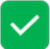
Zheng et al. (77)	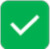	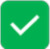	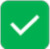	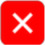	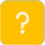	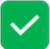	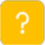	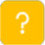	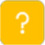	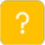	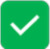
Zisi et al. (78)	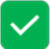	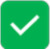	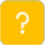	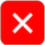	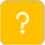	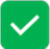	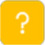	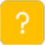	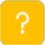	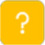	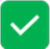
Zwierko (79)	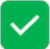	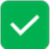	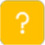	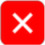	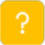	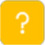	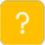	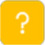	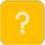	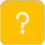	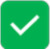
Zwierko (80)	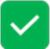	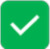	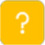	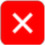	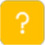	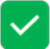	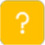	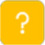	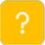	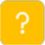	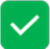
Zwierko et al. (81)	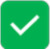	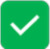	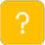	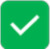	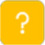	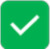	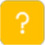	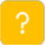	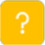	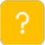	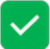
Zwierko et al. (82)	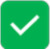	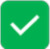	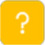	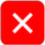	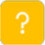	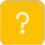	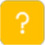	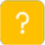	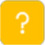	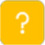	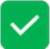
Zwierko et al. (83)	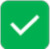	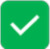	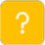	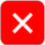	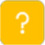	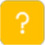	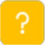	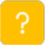	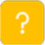	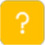	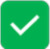
Clinical population											
Chen et al. (14)	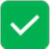	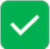	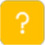	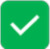	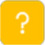	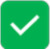	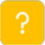	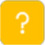	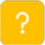	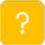	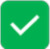
Dong et al. (11)	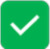	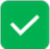	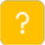	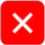	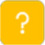	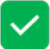	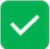	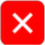	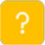	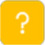	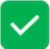
Karakontaki et al. (84)	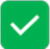	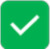	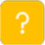	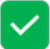	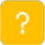	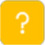	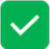	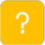	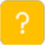	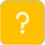	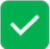
Kim et al. (15)	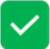	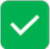	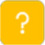	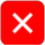	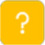	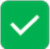	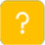	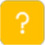	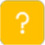	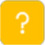	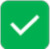
Klasik et al. (16)	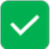	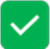	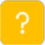	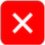	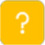	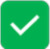	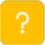	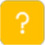	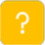	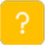	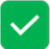
Klasik et al. (12)	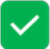	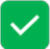	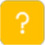	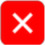	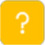	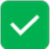	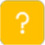	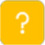	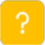	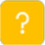	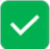
Kloek et al. (85)	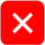	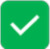	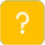	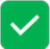	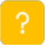	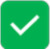	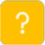	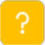	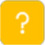	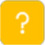	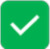
Miah et al. (13)	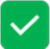	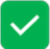	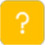	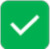	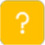	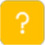	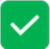	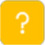	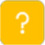	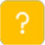	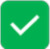
Ramm et al. (86)	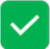	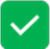	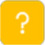	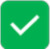	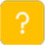	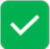	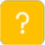	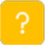	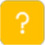	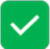	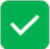
Ramm et al. (87)	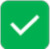	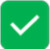	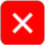	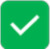	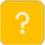	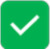	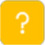	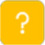	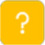	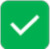	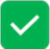
Sabatowski et al. (9)	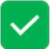	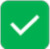	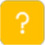	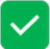	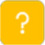	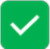	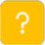	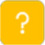	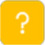	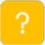	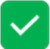
Schmidt et al. (88)	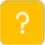	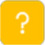	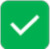	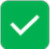	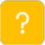	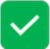	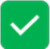	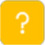	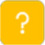	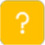	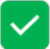
Shmygalev et al. (89)	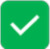	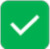	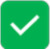	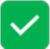	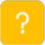	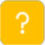	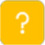	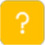	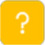	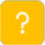	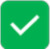
Stubbs et al. (17)	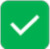	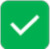	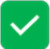	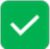	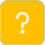	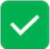	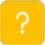	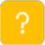	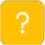	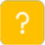	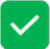

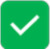
 denotes “Yes”, 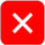 denotes “No”, and 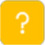 denotes “Unknown”.

**Criterion questions**.

Q1. Was the sample frame appropriate to address the target population?

Q2. Were study participants sampled in an appropriate way?

Q3. Was the sample size adequate?

Q4. Were the study subjects and the setting described in detail?

Q5. Was the data analysis conducted with sufficient coverage of the identified sample?

Q6. Were valid methods used for the identification of the condition?

Q7. Was the data collected by trained observers?

Q8. Were observers similar in terms of clinical experience and level of responsibility?

Q9. When there was more than one observer was comparison of results from across the observers reported?

Q10. Was prevalence reported as a percentage with N or n/N?

Q11. Was a 95% confidence interval or other appropriate measure of variability reported?

#### 3.4.2 According to the scimago journal rank

**Figure 3:**
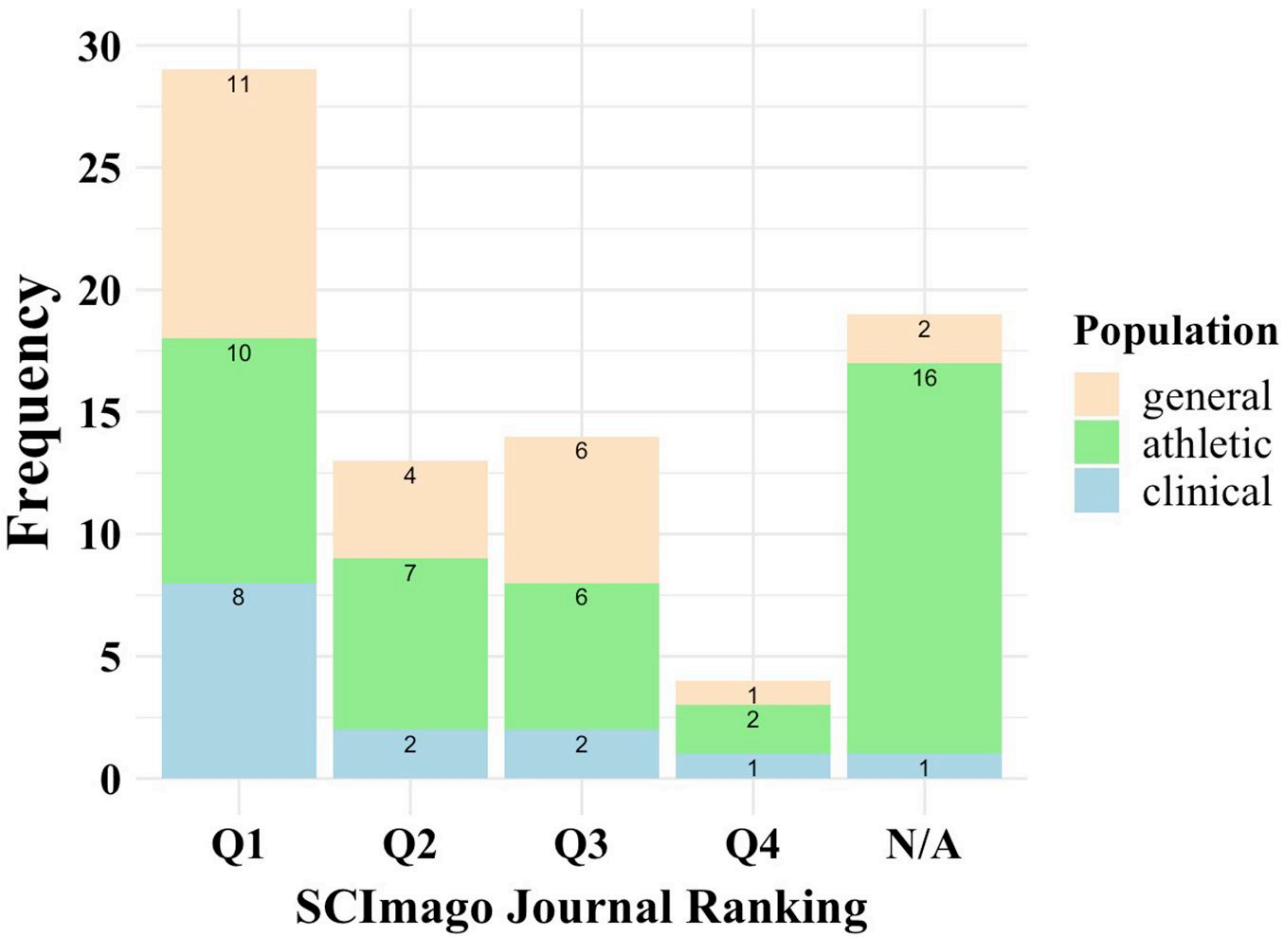
The frequency of articles in the general- (light brown), athletic (green), and clinical (blue) populations in each ranking category of scimago journal rank.

The Scimago Journal Rank (SJR) indicator is a widely accepted measure of scholarly journals' prestige, mostly based on the number and quality of citations. To assess the quality of the articles included in the present global scoping review, we collected each paper's highest SJR in the year of publication. If the study was published in 2024, the previous year's SJR was used. Figure 3 shows the frequency of articles in each population and ranking category. Overall, from the total of 79 articles, 36.7% (*n* = 29) were Q1, from which 11 recruited participants from the general-, 10 from the athletic-, and 8 from the clinical population. This counts for 45.8%, 41.7%, and 57.1% of the total articles in the general-, athletic-, and clinical populations, respectively. From the 16.5% (*n* = 13) of articles with Q2 ranking, 4 (16.7%) recruited participants from the general-, 7 (17.1%) from the athletic-, and 2 (14.3%) from the clinical populations. Regarding articles with Q3 ranking (17.7%, *n* = 14), 6 (25%), 6 (14.6%), and 2 (14.3%) measured cognitive functions with VTS in the general-, athletic-, and clinical populations, respectively. Only 5.1% (*n* = 4) of the articles were Q4; 2 (4.9%) were from the athletic population, and 1 each from the general (4.2%) and clinical (7%) populations. However, 24% (*n* = 19) of the articles don’t appear in SJR. From this, 16 (39%) articles were in the athletic population, and 2 (8.3%) and 1 (7%) in the general and clinical populations, respectively.

Overall, both quality assessments support the idea that many included studies have relatively poor experimental designs, lack a control group, or inconsistently use VTS tests, making it difficult to draw clear conclusions about their validity, feasibility, and reliability. This highlights the need for higher-quality studies.

## 4 Discussion

This scoping review sought to (1) identify the available evidence on the use of VTS, (2) examine how research is conducted using VTS, and, therefore, (3) draw attention to the challenges in the practical application of the VTS for assessing cognitive functions in general, athletic and clinical populations. The main findings are as follows: (1) VTS tests are used to determine cognitive skills of e.g., air traffic controllers, maritime pilots, police officers, train drivers, yoga-practicing older females, professional athletes, and patients with ADHD, depression, Parkinson's disease or schizophrenia, (2) the TOP 5 most popular tests in the VTS are RT (36.7% of total articles, *n* = 29), DT (35%, *n* = 28), COG (34.2%, *n* = 27), PP (19%, *n* = 15) and LVT (16.5%, *n* = 13), 3) only two studies (2.5%, 2/79) received a modified JBI quality score above 70%, while 9 studies (11.4%, 9/79) scored between 50% and 70% 4) only 36.7% of the articles were published in Q1 journals, while almost one-quarter of the papers (24%) were published in journals that do not even appear in Scimago Journal Ranking indicating relatively poor quality of experimental designs, 4) practitioners, athletes, coaches, and trainers, should reconsider the proper use of VTS to assess cognitive functions of interest.

### 4.1 The origins of some VTS tests are not acknowledged

The VTS is a computerized tool that collects many cognitive tests in a modern shape. However, it is important to note that most tests have been used for decades in psychology. For example, Cognitrone Test (COG) is based on Reulecke's theoretical model that considers concentration as an active state of focused attention, which is mainly based on three variables: (1) energy, (2) function, and (3) precision (93). Second, the “Visual Memory Test” (VISGED) test is often used to assess visual short-term memory. However, given its experimental setup, it does not fall into the three general task classes most commonly used in humans to study this cognitive skill. To the best of our knowledge, the Brook Matrix Task (94), the recall procedure of colored squares (95), and the sequential comparison procedure of colored squares [e.g., change-detection task (96)] are the most often used validated tests. In our previous study (8), we speculated that VISGED can be considered a modified version of the recall procedure for colored squares, constructed primarily based on Kosslyn's theory of visual representation (97) and Hänggi's integrative information processing model (98). Third, the so-called “Time/Movement Anticipation Test” (ZBA) is commonly referred to as a “prediction-motion task” (99, 100). Finally, although the present scoping review found that the DT test is among the most commonly used VTS tests, no detailed information is provided on the exact algorithm by which the software adjusts stimulus presentation speed to the participant's performance level. Overall, because the above-mentioned VTS tests are often modified versions of psychological tests used for decades, there is a need to experimentally investigate whether the results of VTS tests and those of their original versions would strongly correlate, thereby strengthening the validity, feasibility, and reliability of VTS.

### 4.2 Discrepancies in the naming of VTS tests

Inconsistent naming of VTS tests across studies makes it difficult to identify the cognitive tests used. What is more, Baur et al. (46) even called the VTS as “Vienna Reaction Apparatus”. In addition, the name “Visual Pursuit Test” (LVT) is misleading, as “pursuit” refers to tracking moving objects with the eyes; however, this test does not measure eye movement behaviors. In Supplementary Tables S1–S3, we noted when the VTS tests were used incorrectly. For example, LVT is called VPT (42); PERSEV is called VPT (13); ZBA is called SDE (60), TMA (69), or motor-temporal test (79); SIGNAL is called Special Ability Signal test (61, 83); or COG is called Selective Attention Test, and DT is called Permanent Attention Test (84). In some other cases, the naming is still incorrect but can be identified more easily, e.g., PP is called peripheral reaction time (82), ATAVT is called TAVT, and VIGIL is called VIG (89). Finally, some studies (29, 31) did not specify the VTS tests used. Overall, future studies should pay more attention to the accurate naming of VTS tests.

### 4.3 Inappropriate data analyses, questionable results

To assess the quality of the involved articles, a modified version of the JBI critical appraisal checklist was used. Our results revealed that of the 79 included studies, only 2 scored above 70%, while 9 scored between 50% and 70%. One of the main reasons for this is the lack of pre-statistical power analysis to estimate a minimum sample size for adequate precision of the prevalence estimate. Sample size calculation is crucial for publication in reputable, peer-reviewed journals, as it guarantees the study's statistical power to identify genuine effects and enhances methodological rigor. Second, most studies received an “unknown” rating for whether they conducted data analysis with complete coverage of the identified sample and for reporting interobserver comparisons when multiple observers were involved in data collection. Furthermore, many of the identified studies did not report whether the data were collected by trained observers. This must be acknowledged in future studies, given that VTS is based on psychological tests; therefore, an experienced psychologist or other trained professional must be involved in VTS studies. Lastly, only 29 studies described the participants and study setting in detail. Others failed to report demographic data (e.g., gender, age) or key participant characteristics (e.g., years of experience), misused the VTS test, or reported only descriptive statistics.

In addition, we also reported the highest Scimago Journal Ranking of each involved paper as part of the quality assessment. While the journal's quartiles are not a proxy for high-quality research, the results align with the biases and quality issues identified in the JBI quality assessment. Specifically, the findings of the present systematic scoping review revealed that from the total of 79 articles, only 36.7% (*n* = 29) were Q1, from which 11 recruited participants from the general-, 10 from the athletic-, and 8 from the clinical population. The number of publications in the other quartiles is shown in Figure 3. It is important to note that almost one-quarter of the papers (24%) were published in journals that do not even appear in the Scimago Journal Ranking, indicating a relatively poor quality of experimental design or analysis. This scoping review does not aim to criticize previous publications on the topic; nevertheless, we would like to draw the scientific community's attention to some factors that must be omitted in scientific research.

Although inaccurate company naming also appears in some cases, i.e., one study (34) referred to VTS as a company from Australia, not Austria, the largest concerns are related to inappropriate data analyses, which make the results questionable. For example, one study (31) used *t*-tests to compare pretest and posttest results; however, an ANOVA should have been used, given that they had both an experimental and a control group. Another study (50) examined the effects of cognitive training on junior soccer players' cognitive performance; however, the authors did not distinguish between professional and non-professional players in their analyses. Moreover, Johne et al. did not present statistical results (55), Kiss et al. reported only descriptive statistics (59), and Zwierko did not provide *post-hoc* test results (80). In another study, the interpretation of the data is exaggerated: Khani et al. used VTS to test sustained attention but concluded that “intensity of the blows in amateur boxing did not cause brain damage”. Without neuroimaging data, it is highly questionable to draw such a conclusion. Inappropriate data reporting also appeared in some clinical studies. For instance, Klasik et al. (12) did not include a healthy control group, did not describe the interventions in detail, and failed to provide effect sizes. In addition, the interpretation of their results is difficult to understand, given that CORSI values were smallest in GROUP 2 and largest in GROUP 1; however, statistical significance was found only between GROUPS 1 and 3. Finally, publications with many typos and grammatical errors could also be found (68). Overall, while VTS tests seem widely used to assess cognitive skills across different populations, in some cases, it is difficult to determine whether the lack of expected findings is due to the limited potential of VTS tests or to poor experimental design and/or analyses in some studies.

### 4.4 Limitations and future directions

In addition to the previously mentioned factors, other limitations create further challenges in the practical application of the VTS for assessing cognitive functions. First, some commonly used test sets have limitations. For example, as many researchers acknowledge, the VTS TRAFFIC package is not necessarily predictive of actual driving behavior (6). Moreover, the company states that (1) “the SFMOTOR test set emerged from a series of sport psychology studies conducted at the psychology faculty of the University of Vienna…” and (2) “The findings of the studies led to the development of the SFMOTOR test set and definition of a target range for the ability profile of elite racing drivers”, however, we found no peer-reviewed publication of these validation studies. Given that most tests are based on standard psychological tests (95, 97–100), the results may provide useful information about participants' cognitive performance. On the other hand, it is not well described how different tests are allocated to specific test sets. For example, according to the manufacturer's claim, VISGED is “marketed for motorsport”. However, considering that most drivers don't need to look for a taxi or specific locations while racing, its relevance is questionable.

Furthermore, given that the VTS cognitive tests were designed for normal or clinical populations, it remains unknown whether elite athletes would also fall within their scope. In line with this, a previous study (25) also acknowledged the scant evidence concerning VTS's predictive validity in older samples. Finally, although the number of familiarization trials was pre-programmed in the VTS software, we found no data or validation studies to support whether the familiarization approach is appropriate or sufficient. Overall, there is a need to experimentally investigate whether the results of VTS tests and their original versions would strongly correlate, thereby strengthening the validity and feasibility of VTS.

Regarding the limitations of the present scoping review, the most significant is the heterogeneity in experimental designs, participants, and VTS tests across the included studies, which prevented us from conducting a meta-analysis or any other subgroup analyses. Nevertheless, we believe the quality analysis in the current review will accurately highlight the potentials and limitations of VTS. Second, almost one-quarter of the included articles (24%) have not been published in a ranked journal according to the Scimago Journal Ranking, which strongly biased the JBI quality assessment results. However, because the exclusion of such articles was not part of our search strategy, we decided to report them in the present scoping review, highlighting the challenges in applying VTS. Overall, the exploratory nature of our scoping review highlights research gaps in the use of VTS rather than providing definitive answers for its use in clinical practice; however, this knowledge can support future studies in creating more rigorous experimental designs and data presentations.

### 4.5 Perspective

The VTS is a widely used computerized tool for assessing psychology-related constructs in different populations. Indeed, our analysis revealed that of the 79 identified articles, 24 used the VTS to evaluate cognitive function in the general population (e.g., air traffic controllers, maritime pilots, police officers, train drivers, yoga-practicing older females). At the same time, 41 and 14 studies recruited participants from the athletic (e.g., yoga-practicing older females, racing drivers, soccer players, wrestlers and taekwondo competitors, boxers, rhythmic gymnasts) and clinical (e.g., patients with ADHD, depression, Parkinson's disease or schizophrenia) populations, respectively.

Despite the increasing popularity of VTS over the past 2 decades, to the best of our knowledge, only 2 review papers have been published on this topic. While Ong (18) aimed to provide a comprehensive review of the literature on the use of VTS in sports psychology, Vater & Strasburger (19) searched the literature to identify the five most widely used peripheral vision tools in sports, of which VTS is one. In the present scoping review, we sought to provide a global overview not only of the available evidence on the use of VTS but also to draw attention to the challenges in the practical application of the VTS for assessing cognitive functions in general, athletic and clinical populations.

Our findings suggest that a wide spectrum of cognitive tests of the VTS has the potential to assess e.g., reaction time, sustained attention, peripheral perception, stress reactivity, or visual orientation in the general, athletic and clinical populations; however, only 36.7% of the articles were published in Q1 journals, while almost one-quarter of the papers (24%) were published in journals that do not even appear in Scimago Journal Ranking. The relatively poor experimental designs in many VTS studies make it difficult to draw clear conclusions about its validity and feasibility. Therefore, future research should develop more sophisticated experimental designs and explore the full potential of VTS in terms of validity and feasibility.

## 5 Conclusions

The aims of this scoping review were to (1) identify the available evidence on the use of VTS, (2) examine how research is conducted using VTS, and, therefore, (3) draw attention to the challenges in the practical application of the VTS for assessing cognitive functions in general, athletic and clinical populations. The findings from the review highlight some important points: (1) VTS tests are used to determine cognitive skills of e.g., air traffic controllers, maritime pilots, police officers, train drivers, yoga-practicing older females, professional athletes, and patients with ADHD, depression, Parkinson's disease or schizophrenia, (2) the TOP 5 most popular tests in the VTS are RT, DT, COG, PP and LVT, (3) only two studies received a modified JBI quality score above 70%, while 9 studies scored between 50% and 70%, 4) only 36.7% of the articles were published in Q1 journals, while almost one-quarter of the papers (24%) were published in journals that do not even appear in the Scimago Journal Ranking indicating relatively poor quality of experimental designs. Future studies should use the VTS in well-designed experimental setups under well-controlled conditions to improve public perception of its validity, feasibility, and reliability.
