# Correction: Challenges in the practical application of the Vienna test system for assessing cognitive functions in the general, athletic and clinical populations: a global scoping review of experimental and observational studies

**DOI:** 10.3389/fspor.2026.1844421

**Published:** 2026-04-28

**Authors:** János Négyesi, Péter Kovács, András Attila Horváth

**Affiliations:** 1 Department of Kinesiology, Hungarian University of Sports Science, Budapest, Hungary; 2 Neurocognitive Research Center, Nyírő Gyula National Institute of Psychiatry, and Addictology, Budapest, Hungary; 3 CRU Hungary Kft., Budapest, Hungary; 4 Department of Anatomy Histology and Embryology, Semmelweis University, Budapest, Hungary; 5 Faculty of Medicine, Universidade de Lisboa, Lisbon, Portugal

**Keywords:** cognitive skills, patients, sport, traffic, VTS

Author András Attila Horváth was erroneously omitted as a corresponding author.

The original version of this article has been updated.

