# Supplementary material for: Challenges in the practical application of the Vienna test system for assessing cognitive functions in the general, athletic and clinical populations: a global scoping review of experimental and observational studies
Source: Front Sports Act Living. 2026 Feb 23;8:1716584. doi: 10.3389/fspor.2026.1716584 (PMC12968311; doi:10.3389/fspor.2026.1716584)
Supplement: Supplementary file 1 [file Supplementaryfile1.docx]

| **Attachment 1:** Literature search strategy, PRISMA-ScR Flow Diagram (Fig. 1) | |
| --- | --- |
| **DATABASE** | **PubMed, Web of Science, ELSEVIER Scopus, EBSCOhost** |
| **DATE** | **11/09/2024** |
| **STRATEGY** | **(#1 AND #2 AND #3) NOT (#4)** |
| **#1** | ‘Vienna Test System’ OR ‘VTS’; TI/AB |
| **#2** | ‘cognition’ OR ‘cognitive’ OR ‘cognitive function’ OR ‘global cognition’ OR ‘executive function’ OR ‘executive cognition’ OR ‘memory’ OR ‘memory function’; TI/AB |
| **#3** | ‘randomized controlled trial’ OR ‘controlled clinical trial’ OR ‘randomized’ OR ‘placebo’ OR ‘control’ OR ‘clinical trials’ OR ‘randomly’ OR ‘trial’; TX |
| **#4** | ‘cell’ OR ‘cellular’ OR ‘molecular’; TI/AB |
| *AB, Abstract; TI, Title; TX, All Text; AND/OR/NOT= Boolean Operators* | |
