# Supplementary material for: Challenges in the practical application of the Vienna test system for assessing cognitive functions in the general, athletic and clinical populations: a global scoping review of experimental and observational studies
Source: Front Sports Act Living. 2026 Feb 23;8:1716584. doi: 10.3389/fspor.2026.1716584 (PMC12968311; doi:10.3389/fspor.2026.1716584)
Supplement: Supplementary file 2 [file Table1.docx]

| **Supplementary Table 1:** The application of Vienna Test System for assessing cognitive function in the general population | | | | | | | | | | | | | |
| --- | --- | --- | --- | --- | --- | --- | --- | --- | --- | --- | --- | --- | --- |
| **Study** | **SJR** |  | **Participants characteristics** |  | **Study objectives** |  | **Intervention** |  | **VTS cognitive tests** |  | **Key VTS-related findings** |  | **Remarks** |
| Aporosa  et al 2020 (57) | Q3 |  | active kava drinkers  (n=20, age: 35.3±9.5y,  2 females)  control non-drinkers  (n=20, age: 35.1±10.4y,  2 females) |  | To measure selected cognitive functions following high traditionally consumed kava volumes. |  | 6-hr kava session in a “naturalised” setting |  | WAF-A (reaction and attention intensity)  WAF-G (parallel processing and selectivity of attention) |  | non-significant differences between groups |  | Not a well-controlled study. |
| Balzarotti  et al 2022 (58) | Q2 |  | healthy old drivers (n=58, age: 82.8±6.1y,  2 females) |  | To examine whether older drivers’ cognitive performances declined from the first to the second administration. |  | N/A |  | RT (decision time and motor time)  DT (reactive performance)  COG (selective attention)  ATAVT (visual orientation, perceptual speed) |  | RT  T1 < T2  (consistent with practice effects)  COG  T1 > T2  RT, DT, COG, ATAVT performance  restricted < unrestricted |  | Scant evidence exists concerning VTS’s predictive validity in older samples. |
| Bartolacci  et al 2020 (6) | Q3 |  | healthy young adults  (n=40, age: 26.2±3.5y, 19 females)  healthy older adults  (n=40, age: 66.6±5.6y, 16 females) |  | To investigate the influence of sleep quality, vigilance, and sleepiness on driving-related cognitive abilities in younger and older adults. |  | N/A |  | **TRAFFIC package:**  COG (selective attention)  ATAVT (visual orientation, perceptual speed)  WRB-TV (subjectively accepted risk level) |  | COG  older > young  1) response time in correct rejection  2) working time  ATAVT  1) older > young  working time  2) older < young  performance |  | The sleep–wake schedule was not controlled.  The VTS TRAFFIC package is not necessarily predictive of the actual driving behavior. |
| Deng & Wu 2020 (44) | Q2 |  | introverts  (n=15, 5 females)  extraverts  (n=15, 7 females) |  | To investigate the impact  of background music on cognitive performance  of introverts and extraverts. |  | performing cognitive tasks in silence and with  Chinese pop as background music |  | RT (decision time and motor time)  LVT (visual orientation, attention) |  | RT  non-significant differences between groups  LVT  1) silence > music  2) extraverts > introverts (music) |  | Demographic data of participants is not provided.  Low sample size. |
| Grundler & Strasburger 2020 (7) | Q1 |  | older drivers  (n=84, age: 68.9±6.6y, 28 females) |  | To determine whether VTS could predict older adults’ on-road driving performance. |  | N/A |  | **Expert System Traffic package:**  AMT (intelligence)  NVLT (learning ability)  B19 (visuo-motor coordination)  TAVTMB (PercPer in traffic)  DT (reactive performance)  RT (decision time and motor time)  COG (selective attention)  SIGNAL (selective attention)  PP (divided attention, peripheral detection) |  | VTS is excellent in identification of good drivers  VTS failed in the prediction of bad driving performance  visual performance indicators were less indicative of driving ability than psychometric assessment  selective and divided attention turned out to be much more important for predicting fitness to drive than either visual acuity, size of the visual field, or contrast sensitivity |  | The sample was pre-screened for clear-cut visual deficits; thus, it may not fully represent the population of older drivers. |
| Hani Tabai  et al 2018 (43) | Q1 |  | drivers without accident  (n=34, age: 38.9±7.8y)  drivers with accident  (n=22, age: 39.6±5.5y) |  | To investigate the effects of train drivers’ cognitive and demographic characteristics on the occurrence of rail accidents. |  | N/A |  | WAF-V (perception and attention)  COG (selective attention)  LVT (visual orientation, attention) |  | the sustainable attention of train drivers is one of the predictors of rail crashes  non-significant relationship between the demographic characteristics and occurrence of the accidents |  | Gender of participants is not provided. |
| Hedayati  et al 2021 (51) | Q1 |  | air traffic controllers  with a history of  human error  (ERR, n=17,  only males)  without a history of  human error (ERRFREE, n=20,  only males) |  | To investigate the effect of cognitive functions (and personality traits) on the occurrence of human error in air traffic controllers. |  | N/A |  | MR (spatial perception)  CORSI (visual-spatial memory)  DAUF (sustained attention)  TOL-F (planning ability) |  | MR, DAUF  ERRFREE > ERR  CORSI, TOL-F  ERRFREE = ERR |  | Demographic data of participants is not provided.  Not a well-controlled study.  (causal-comparative method) |
| Jiménez-Pavón et al 2011 (52) | Q3 |  | physically active adults  (n=16, age: 21.1±1.4y, mass: 74.3±6.6kg, height: 178±4cm,  only males) |  | To examine the effect of a running bout under hot conditions on cognitive performance in physically active men. |  | running at 60% of maximum aerobic speed for ~1hour under hot (35°C, 60% rel. humidity) environmental conditions |  | RA (simple, choice, multiple, and peripheral vision reaction time)  number of correct/incorrect responses  field of vision |  | 1) T1 < T2  choice reaction time  multiple reaction time  peripheral reaction time  number of incorrect responses  2) T1 > T2  right visual angle |  | The exact names of the used VTS tests are not provided. |
| Johnsen  et al 2024 (53) | Q1 |  | police officers  (n=129, age: 25–45y,  15 females) |  | To evaluate the cognitive tests used in the selection procedure for applicants to an educational program for Norwegian police drone  pilots and their relation to performance during the simulated flight. |  | N/A |  | A3DW (spatial perception)  INT (logical reasoning)  TACO (attentional selection)  VIGIL (sustained attention)  VISGED (visual short-term memory) |  | low-to-moderate intercorrelations of the cognitive tests  only A3DW, INT, and TACO were correlated to the performance measures of skills and proficiency  only A3DW and TACO had unique contributions in explaining the variance in both performance measures |  | The study did not control potential influencing variables (e.g., skills in video games). |
| Laux & Corazza 2019 (54) | Q4 |  | physically inactive subjects  EXP  (n=13, age: 29.5±7.5y, mass: 74±18.3kg, height: 168±0cm,  7 females)  CON  (n=13, age: 29.8±5.6y, mass: 73.7±16.1kg, height: 167±0cm,  8 females) |  | To verify the effects of a workplace physical exercise program on the simple and choice reaction times of public university employees. |  | 36 sessions with physical exercises in the workplace, held 3d/w for 12w |  | SRT (simple reaction time)  CRT (choice reaction time)  movement time  response time |  | T1 > T2 (EXP)  reaction time  movement time  response time |  | Incorrect statistical analyses (ANOVA must have been applied). The results are questionable.  The exact names of the used VTS tests are not provided. |
| Loch  et al 2020 (45) | Q2 |  | undergraduate and graduate students  (n=24, age: 22.8±3.6y, 14 females) |  | To examine acute effects of potential mental recovery strategies (MR) on subjective-psychological and on cognitive performance outcomes after a mentally fatiguing task. |  | 4 consecutive intervention sessions (powernap [PN], a systematic breathing [SB], SB+mental imagery [SB+], and a control condition [CC]), 1d/w |  | SRT (simple reaction time)  motor time |  | T1 > T2  SRT, motor time |  | In the participants’ pre-experiment states, no initial cognitive performance test was administered. |
| Malik  et al 2024 (59) | Q1 |  | physically active older adults  (n=26, age: 70.1±4.4y, 21 females) |  | To investigate the effect of a juggling intervention on postural stability and selected cognitive abilities in active older adults. |  | juggling period (JP)  3d/w for 4w  control phase (NJP)  3d/w for 4w |  | SRT (simple reaction time)  CRT (choice reaction time)  COG (selective attention) |  | T1 > T2 (JP, NJP)  SRT, COG |  | A larger sample would have been desirable to obtain stronger results (medium effect sizes). |
| Mehri  et al 2023 (46) | Q3 |  | students  (n=35, age: 26.1±2.1y, only males) |  | To determine the effect of traffic noise on cognitive functions. |  | exposure to recorded traffic noise for 1 hour |  | PP (divided attention, peripheral detection)  ATAVT (visual orientation, perceptual speed) |  | silent < noise (introvert)  ATAVT |  | VTS is referred as a company from Australia; ATAVT is written as ATTPT, etc. |
| Mikicin 2021 (47) | Q3 |  | students  EXP  (n=30, only males)  CON  (n=10, only males) |  | To psychologically assess the overall ability to be attentive during the prolonged focus of oriented visual perception during task performance. |  | neurofeedback training  20x30min, 1/week |  | COG (selective attention)  DAUF (sustained attention)  LVT (visual orientation, attention) |  | 1) COG  time of correctly accepted stimuli: T1 > T2  2) DAUF  sum of correct responses: T1 < T2  time of correct and incorrect responses:  T1 > T2  3) LVT  median time of correct responses: T1 > T2  score based on consideration time:  T1 < T2 |  | Demographic data of participants is not provided.  Low sample size.  Only males were involved. |
| Mudrák & Slepicka 2014 (60) | N/A |  | seniors  (n=33, age: 60–92y,  23 females) |  | To examine the relationship between the age-related cognitive decline and decline in cognitive processing speed. |  | N/A |  | DT (reactive performance)  COG (selective attention)  RT (decision time and motor time)  LVT (visual orientation, attention)  VISGED (visual short-term memory) |  | 1) age ~ RT  variance (+)  2) age ~ LVT  score (-)  median time for correct answers (+)  working time (+)  3) age ~ DT  median reaction time (+)  number of stimuli (-)  correct answers (-) |  | Not a well-designed study. |
| Müller et al 2013 (48) | Q1 |  | 6th grade students  SL-HL  (n=49, age: 12.6±0.5y, 28 females)  HL-SL  (n=56, age: 12.6±0.6y, 27 females) |  | To examine the impact of skipping lunch (SL) vs having lunch (HL) on children’s cognitive functioning in the early afternoon. |  | N/A |  | WAF-A (reaction and attention intensity)  CORSI (visual-spatial memory)  COG (selective attention) |  | SL > HL  WAF-A (omitted errors) |  | Not a well-controlled study. |
| Niedzielska et al 2023 (49) | Q3 |  | physical education students  (n=29, age: 19-25y,  20 females) |  | To compare the effectiveness of performing tasks involving different cognitive functions in sitting versus standing positions. |  | N/A |  | COG (selective attention)  ALS (concentration, saturation and mental fatigue) |  | non-significant differences between positions |  | N/A |
| Roman-Liu & Mockałło 2020 (61) | Q1 |  | older individuals  (n=26, age: 68±2.9y,  12 females) |  | To determine the impact bimanual coordination tasks performance on coordination skills and cognitive functions. |  | 6 sessions of bimanual coordination training |  | COG (selective attention)  RT (decision time and motor time) |  | T1 < T2  RT (speed of movement in planned action sequences) |  | Neurocognitive diseases were not controlled. |
| Saadat et al 2018 (50) | Q1 |  | medical students  (n=22, age: 18–22y,  only males) |  | To find out whether there is a causal relationship between waterpipe smoking (WPS) and driving performance. |  | waterpipe smoking after breakfast |  | ATAVT (visual orientation, perceptual speed)  COG (selective attention)  DT (reactive performance)  LVT (visual orientation, attention)  PP (divided attention, peripheral detection)  ZBA (speed and movement in space) |  | T1 > T2  COG  T1 < T2  DT (correct answers) |  | Only short-term effects were investigated. |
| Seyfzadeh-  darabad et al 2023 (63) | Q1 |  | Iranian maritime pilots  (n=72, age: 28-66y,  only males) |  | To explore the relevance of cognitive deficits to human error occurrence in  maritime operations. |  | N/A |  | CORSI (visual-spatial memory)  WAF-F (focused attention)  WAF-G (parallel processing and selectivity of attention)  MR (spatial perception)  TOL-F (planning ability)  ZBA (speed and movement in space) |  | no history of error > error  CORSI  WAF-G  MR |  | Did not consider the role of circadian rhythm. |
| Tabai et al 2018 (55) | Q1 |  | train drivers  (n=56, age: 39.2±6.9y,  only males) |  | To investigate the effects of train drivers’ cognitive and demographic characteristics on the occurrence of rail accidents. |  | N/A |  | WAF-V (perception and attention)  COG (selective attention)  LVT (visual orientation, attention) |  | without accident > accident  WAF-V |  | N/A |
| Taheri & Irandoust 2017 (62) | N/A |  | older individuals  (n=29, age: 63-71y,  only females) |  | To investigate the effect of balance and computerized cognitive training on psychomotor performance in older females |  | 1) balance training 3 d/w for 12w  2) balance training with COG  3 d/w for 12 w  3) COG  4) CON |  | DT (reactive performance)  LVT (visual orientation, attention) |  | T1 < T2  DT, LVT (each group) |  | Incorrect naming of the VTS test (LVT is called VPT). Inaccurate presentation of statistical results. |
| Tinella et al 2020 (64) | Q2 |  | young healthy participants  (n=100, age: 23.1±3.6y, 33 females)  older healthy participants  (n=83, age: 54.1±7.3y, 30 females) |  | To investigate the relationship between overall cognitive functioning, self and object-based spatial mental transformation skills, and driving performance in a sample of younger and older adult drivers. |  | N/A |  | RT (decision time and motor time)  DT (reactive performance)  ATAVT (visual orientation, perceptual speed) |  | 1) young > older  RT, DT, ATAVT  2) cognitive functioning predicted traffic stress resilience  3) self and object-based spatial mental transformation skills affect stress resilience |  | N/A |
| Yeo et al 2012 (56) | Q1 |  | healthy individuals  (n=15, age: 19-25,  only males) |  | To investigate the effect of Korean red ginseng on cognitive performance. |  | GIN (n=8)  4500mg/d for 2w  CON (n=7)  placebo for 2w |  | VIGIL (sustained attention)  RT (decision time and motor time)  MLS (fine motor abilities) |  | non-significant differences between groups |  | Low sample size, potentially low dose of ginseng, and short trial duration. |
| A3DW: Adaptive Spatial Ability Test, ALS: Work Performance Test, AMT: Adaptive Matrices Test, ATAVT: Adaptive Tachistoscopic Traffic Perception Test, B19: Double Labyrinth Test, COG: Cognitrone Test, CON: control group/condition, CORSI: Corsi-Block-Tapping-Test, DAUF: Continuous Attention Test, DT: Determination Test, EPP6: Eysenck Personality Profiler Test Version 6, EXP: experimental group, INT: Logical Reasoning, LVT: Visual Pursuit Test, MLS: Motor Performance Series, MR: Mental Rotation Test, NVLT: Non-Verbal Learning Test, PercPer: Perceptual Performance, PP: Peripheral Perception Test, RA: Reaction Time Analysis, RT: Reaction Test, SJR: Scimago Journal Ranking, SIGNAL: Signal Detection, T: Time, TAVTMB: Tachistoscopic Traffic Test, TACO: Attention and Concentration Test, TOL-F: Tower of London Test - Freiburg Version, VIGIL: Vigilance Test, VISGED: Visual Memory Test, WAF: Perception and Attention Functions Battery, WRB-TV: Vienna Risk-Taking Test Traffic, ZBA: Time/Movement Anticipation | | | | | | | | | | | | | |
