# Supplementary material for: Challenges in the practical application of the Vienna test system for assessing cognitive functions in the general, athletic and clinical populations: a global scoping review of experimental and observational studies
Source: Front Sports Act Living. 2026 Feb 23;8:1716584. doi: 10.3389/fspor.2026.1716584 (PMC12968311; doi:10.3389/fspor.2026.1716584)
Supplement: Supplementary file 3 [file Table2.docx]

| **Supplementary Table 2:** The application of Vienna Test System for assessing cognitive function in the athletic population | | | | | | | | | | | | | |
| --- | --- | --- | --- | --- | --- | --- | --- | --- | --- | --- | --- | --- | --- |
| **Study** | **SJR** |  | **Participants characteristics** |  | **Study objectives** |  | **Intervention** |  | **VTS cognitive tests** |  | **Key VTS-related findings** |  | **Remarks** |
| Baláková  et al 2015 (79) | Q2 |  | Czech youth soccer players  (n=91, age: 13 y,  only males) |  | To determine the relationship between talent and achievement variables in the Vienna Test System. |  | N/A |  | SPM (general intelligence)  RT (decision time and motor time)  DT (reactive performance)  LVT (visual orientation, attention)  CORSI (visual-spatial memory)  ZBA (speed and movement in space)  PP (divided attention, peripheral detection) |  | talented > non-talented  ZBA (deviation of movement median) |  | The talented group attained significantly better results on only 1 of the 16 variables. Therefore, the practical significance of VTS tests for talent identification is questionable. |
| Baur et al. 2006 (26) | Q1 |  | elite racing drivers  (n=8, age: 26.5±5y, mass: 68.8±7.8kg, height: 1.77±0.04m, only males)  control group  (n=10, age: 26.2±6y, mass: 72.7±7.5kg, height: 1.81±0.08m, only males) |  | To compare reaction time, stability performance capacity, and strength performance capacity of elite racing drivers with those of age-matched, physically active controls. |  | N/A |  | RT (decision time and motor time)  DT (reactive performance) |  | drivers < controls  RT |  | Low sample size. |
| Blecharz et al 2022 (65) | Q2 |  | handball players  (n=50, age: 22.4±4.7y) |  | To determine the level of selected cognitive traits in handball players while considering their sports level and the assigned position on the court. |  | N/A |  | STROOP (ability to inhibit overlearned answers)  CORSI (visual-spatial memory)  PP (divided attention, peripheral detection)  RT (decision time and motor time) |  | goalkeepers < other players  STROOP, CORSI |  | Gender is not presented.  The results showed a great variability among handball players. |
| de Andrade et al 2021 (80) | Q1 |  | Brazilian soccer players  (n=80, age: 13.9±1.1y, only males) |  | To examine whether there are differences in the peripheral perception of young soccer players with similar formal practice time and competitive levels, according to their tactical behaviour efficiency. |  | N/A |  | PP (divided attention, peripheral detection) |  | tactically more efficient players < tactically less efficient players  PP reaction time, PP tracking deviation  tactically more efficient players > tactically less efficient players  PP visual field |  | Not a well-controlled study. |
| de Sousa Pinheiro et al 2022 (81) | Q3 |  | Brazilian elite soccer players  1) U15  (n=139, age: 14.2±0.7y, mass: 61.8±7.4kg, height: 1.73±7.1m,  only males)  2) U17  (n=97, age: 18±0.5y, mass: 68.9±7.4kg, height: 1.78±7.4m,  only males) |  | To compare the discriminative reaction time in elite young soccer players between categories U-15 and U-17. |  | N/A |  | RT (decision time and motor time) [S5 - discriminative reaction time] |  | U17 < U15  RT – S5 |  | The effect size is small; therefore, the practical significance is questionable. |
| Fózer-Selmeci et al 2019 (82) | Q4 |  | Hungarian soccer players  (n=63, age: 15-19y,  only males) |  | To examine the effects of cognitive training on junior soccer players’ cognitive  performance. |  | Training (TRN)  Cognitive Training Program (CogniPlus)  2x15-min/w for 12w  Control (CON)  no intervention |  | MDT (cognitive reaction time)  DT (reactive performance)  STROOP (ability to inhibit overlearned answers)  CORSI (visual-spatial memory) |  | DT incorrect  TRN: T1 < T2  CON: T1 ≂ T2 |  | Not a well-designed study, e.g., the authors did not distinguish between professional and non-professional soccer players. |
| Gierczuk & Ljach 2012a (28) | Q3 |  | Polish students  (n=15, age: 16.5±0.5y, only males) |  | To evaluate the accuracy of VST computer tests used in wrestling to evaluate motor skill development. |  | N/A |  | RT (decision time and motor time)  DT (reactive performance)  SIGNAL (selective attention)  2HAND (visual-motor coordination)  MLS (fine motor abilities) |  | reliability and validity are significant at least one of the measures:  RT, DT, SIGNAL, 2HAND, MLS |  | Research in this field is not well-explored. |
| Gierczuk et al 2012b (27) | N/A |  | wrestlers  (n=25, age: 21.9±2.7 y)  ITF taekwondo competitors  (n=24, age: 22.7±5.8y) |  | To compare coordination motor abilities in elite wrestlers and taekwondo competitors. |  | N/A |  | RT (decision time and motor time)  DT (reactive performance)  SIGNAL (selective attention)  MLS (fine motor abilities) |  | wrestlers vs. taekwondo  non-significant differences  wrestlers, taekwondo  higher sports classes were better in RT, DT, SIGNAL, MLS |  | Gender is not presented. |
| Horváth et al 2022 (8) | Q1 |  | car arcing drivers  EXP  (n=12, age: 24.7±4.4y, mass: 79.3±10.9kg, height: 1.81± 0.74kg, only males)  CON  (n=12, age: 25.6±3.3y, mass: 78.8±9.4kg, height: 1.79± 0.92kg, only males) |  | To determine the effects of a 6-week reactive agility training program using light-based stimuli on car racing drivers’ physiological and cognitive abilities |  | EXP  reactive agility training program (Witty SEM light stimulus)  2x60min/w for 6w  CON  no intervention |  | **SFMOTOR package:**  LVT (visual orientation, attention)  STROOP (ability to inhibit overlearned answers)  VISGED (visual short-term memory)  ZBA (speed and movement in space)  DT (reactive performance) |  | 1) LVT  correct answers  T1 < T2  reaction time  T1 > T2  EXP: T1 > T2  CON: T1 ≂ T2  2) STROOP color-naming  incorrect answers  EXP: T1 > T2 (vs. CON)  reaction time  CON: T1 < T2 (vs. EXP)  3) STROOP word-reading  incorrect answers  EXP: T1 ≂ T2  CON T1 > T2  reaction time  EXP: T1 < T2  CON: T1 ≂ T2  4) DT  correct answers  EXP: T1 < T2  CON: T1 > T2  omitted answers  EXP: T1 > T2  CON: T1 ≂ T2 |  | Lack of control group with an alternative reactive agility training regime.  The study carefully points out the limitations of VTS. |
| Horváth et al 2023 (69) | Q1 |  | EXP1 (nM)  (n=11, age: 22.6±3.8y,  2 females)  EXP2 (NT)  (n=10, age: 20.5±1.7y,  1 female)  CON  (n=10, age: 20.3±1.2y,  1 female) |  | To investigate the efficacy of a novel neurofeedback system, called neuroMoon (nM) on cognitive abilities by examining the differences between the improvements after nM vs. NeuroTracker (NT) training. |  | computer-based cognitive training programs  3x/w for 4w  using  1) nM (EXP1)  2) nM sham (CON)  3) NT (EXP2) devices |  | TMT-L (visuomotor processing speed and cognitive flexibility)  STROOP (ability to inhibit overlearned answers)  RT (decision time and motor time)  SWITCH (flexible task-switching ability)  DT (reactive performance) |  | 1) STROOP  median reaction time  T1 > T2  2) SWITCH  working time and mean reaction time  T1 > T2  3) DT  correct answers  T1 < T2  omeitted answers  T1 > T2 |  | Lack of inactive control group.  Relatively low sample size. |
| Huzarska et al 2023 (83) | Q3 |  | young dancers  (n=31, age: 13-16y,  only females) |  | To investigate the relationship between perfectionism and motor-cognitive abilities. |  | N/A |  | ZBA (speed and movement in space)  3D (spatial orientation)  2HAND (visual-motor coordination)  PP (divided attention, peripheral detection) |  | Negative perfectionism is associated with  ZBA |  | Age reporting is misleading.  Certain parameters (MEDDFK, MEDTFE and MEDTFG) are not defined. |
| Johne et al 2013 (30) | N/A |  | female épée fencers  Group 1 (champ. class)  (n=15, age: 21.9±3.5y, mass: 65.1±7.1kg, 1.76±0.66m)  Group 2 (first class)  (n=15, age: 18.1±2.3y, mass: 60.7±6.9kg, 1.74±0.57m)  Group 3 (second class)  (n=15, age: 15.5±1.3y, mass: 57.1±6.5kg, 1.68±0.53m) |  | To assess dynamic asymmetry in terms of complex reaction time in female épée fencers of different sports classes. |  | N/A |  | RT (decision time and motor time)  [S5 - discriminative reaction time] |  | RT  Group 1 < 2 < 3 |  | Statistical results are not reported. |
| Kapur & Joshi 2024 (66) | Q2 |  | fast ball athletes  (n=20, age: 22.1± 2.4y, mass: 65.2±8.9kg, height: 1.7±0.08m) |  | To examine the effects of High Intensity Interval Exercise (HIIE) and Moderate Intensity Continuous Exercise (MCE) on executive function. |  | HIIE  4x4min at 90–95% HRmax with 3 min active recovery at 70% HRmax  MCE  volume-matched to the HIIE  60% of HRmax for 40 min |  | COG (selective attention)  S4 (fixed working time) |  | COG  total correct reaction  T1 < T2, T1 < T3  total incorrect reaction  T1 > T2, T1 > T3  total incorrect non-reaction  T1 > T2, T1 > T3  time of correct reaction  T1 > T2, T1 > T3 |  | Gender is not presented.  Exact name of the VTS test (COG) was not provided. |
| Khani et al 2012 (70) | N/A |  | EXP (4y of experience)  (n=30, age: 21.0±3.1y, only males)  CON (1y of experience)  (n=30, age: 25.0±4.2y, only males)  RUN (400m, 800m)  (n=30, age: 24.0±2.7y, only males) |  | To observe attention impairments in experienced and novice amateur boxers. |  | N/A |  | DAUF (sustained attention) |  | non-significant differences between groups |  | The conclusion from the study results is questionable: ‘intensity of the blows in amateur boxing did not cause brain damage’. |
| Kiss & Balogh 2019 (71) | Q3 |  | competitive handball players  (n=92, age: 19.3±5y,  47 females) |  | To examine the skills of handball players and study the differences between age groups, gender groups and competitive levels. |  | N/A |  | DT (reactive performance)  COG (selective attention) |  | females > males  omitted, reactions, number of stimuli  adult > youth  reactions, incorrect answers, reaction time |  | Effect size is not provided. |
| Kiss et al 2020 (72) | Q3 |  | referees of European Handball Federation  (n=28, age: 22.8±2.8y,  8 females) |  | To study cognitive skills among young handball referees. |  | N/A |  | DT (reactive performance)  COG (selective attention)  LVT (visual orientation, attention) |  | no statistical results are provided |  | Only descriptive statistics were performed. |
| Kunrath et al 2020 (10) | Q1 |  | university first-team soccer players  (n=18, age: 21.8±2.5y, only males) |  | To examine how mental fatigue influences peripheral perception (PP), tactical behaviour, and physical performance of soccer players during a standard small-sided game. |  | cognitive training program:  modified Stroop task and VTS |  | PP (divided attention, peripheral detection) |  | PP (visual field)  T1 > T2 |  | Changes observed in the study may have been the result of prolonged mental effort prior to the field task. |
| Kutlu et al 2023 (73) | N/A |  | professional and amateur billiards players  (n=33, age: 35.3±10.2y, only males) |  | To reveal differences between the cognitive skills between different grade billiards players. |  | N/A |  | DT (reactive performance)  SPM (general intelligence)  RT (decision time and motor time)  ZBA (speed and movement in space) |  | professional > amateur  DT (reaction time)  ZBA (accuracy)  RT ~ billiard scores |  | Incorrect naming of the VTS test (ZBA is called SDE). |
| Lesiakowski et al 2013 (74) | N/A |  | EXP (elite Polish amateur boxers)  (n=15, age: 20.4±5.2y, 5 females)  CON (nonathletes)  (n=15, age: 21.1±2.0y, 5 females) |  | To investigate the visuospatial attentional functioning in amateur boxers vs. nonathletes. |  | N/A |  | SIGNAL (selective attention) |  | non-significant differences between groups |  | Incorrect naming of the VTS test (SIGNAL is called Special Ability Signal test). |
| Liu et al 2018 (75) | N/A |  | expert rifle shooters  (n=5, only females) |  | To optimize focused attention in expert rifle shooters with the use of  neurofeedback training tools and to enhance shooting performance. |  | 6x10min neurofeedback training |  | DAUF (sustained attention) |  | no statistical results are provided |  | Demographic data of participants is not provided.  Very low sample size. |
| Mikicin &Szczypińska 2021 (76) | Q2 |  | handball players  (n=10, only females) |  | To examine if and to what extent a perceptual-motor training improves sensorimotor coordination, peripheral perception, and general attention in handball players. |  | perceptual-motor training program  2/w for 10w |  | PP (divided attention, peripheral detection)  SMK (sensomotor coordination ability)  COG (selective attention) |  | T1 < T2  SMK  time in ideal area %  time in ideal area % after 5 minutes  T1 > T2  COG  mean time of correctly rejected answers |  | Demographic data is not provided.  Relatively low sample size. |
| Nederhof et al 2007 (31) | Q1 |  | well-trained cyclists  (n=14, age: 25.3±4.1y, 4 females)  CON  (n=14, age: 25.4±4.6y, 5 females) |  | To test the psychomotor slowness hypothesis studying the effects of high load training. |  | high load training  (regular training camp) |  | DT (reactive performance) |  | non-significant differences between groups |  | N/A |
| Nederhof et al 2008a (32) | Q2 |  | Dutch varsity rowers  (n=26, age: 21.3±1.6y, 12 females) |  | To determine whether psychomotor speed is sensitive to changes in perceived performance in a normal rowing season. |  | five occasions during the rowing season |  | DT (reactive performance) |  | DT (reaction time) ~ perceived performance |  | It was not possible to adequately assess actual performance. |
| Nederhof et al 2008b (33) | Q1 |  | female speed skaters  (n=3)  1) nonfunctional overreached (NFO) athlete (age: 16y, mass: 53kg, height: 1.68m)  2) recovering NFO athlete (age: 19y, mass: 69kg, height: 1.74m)  3) Control (age: 17y, mass: 53kg, height: 1.69) |  | To report RESTQ-sport and POMS scores, reaction times, and cortisol and ACTH reactions to a double exercise protocol in three cases, a healthy athlete and two athletes who consulted a sports physician with complaints of underperformance and fatigue. |  | double exercise protocol |  | DT (reactive performance) |  | DT  median reaction time  NFO > CON > recovering NFO |  | Sample size of 3.  The Vienna Determination Test and the double exercise protocol seem to be promising tools for the diagnosis of NFO. |
| Pahan & Singh 2022 (68) | N/A |  | sports trainees  (n=16, age: 11-14y,  only males) |  | To examine how the cognitive performances of preadolescent sports trainees living and training under a controlled environment are affected during the three different day times. |  | N/A |  | SIGNAL (selective attention)  RT (decision time and motor time)  AMT (non-verbal general intelligence)  DT (reactive performance)  LVT (visual orientation, attention) |  | non-significant differences between time points |  | The relevancy of the study goal is questionable. |
| Sadowski et al 2012 (34) | N/A |  | elite taekwondo competitors  (n=63, age: 15.9±0.8y, only males) |  | To identify selected success factors of elite Olympic taekwondo competitors in the context of medals that they have or have not won during Polish Junior Championships. |  | N/A |  | RT (decision time and motor time)  DT (reactive performance) |  | complex reaction time had the largest influence on the number of points scored (16%) |  | Many typos and grammatical errors can be found within the paper.  Lack of control group (another sport or inactive population). |
| Schumacher et al 2018 (87) | Q1 |  | highly talented soccer players  (n=178, age: 10-33, only males) |  | To determine the relation of age and position to general perceptual-cognitive abilities. |  | N/A |  | ZBA (speed and movement in space)  DAUF (sustained attention) |  | DAUF  number of correct responses  U12, U13 < U16-U23, professional team (PT)  percentage of errors  U13 > PT |  | Incorrect naming of the VTS test (ZBA is called TMA, and probably DAUF is called CCT). |
| Szczypińska & Mikicin 2019 (77) | Q3 |  | Polish handball players  (n=18, 9 females) |  | To analyse the changes in the level of cognitive skills in handball players following neurofeedback-EEG trainings. |  | neurofeedback training with EEG DigiTrack Biofeedback system  1-2/w for 20x |  | PP (divided attention, peripheral detection)  SMK (sensomotor coordination ability)  COG (selective attention) |  | T1 < T2  PP (visual field)  SMK (time in ideal area %, time in ideal area % after 5 minutes)  COG (sum of correctly accepted answers) |  | Demographic data is not provided.  Relatively low sample size. |
| Szwarc et al 2021 (84) | Q2 |  | soccer players  (n=64, age: 11-14,  only males) |  | To determine the prognostic value of the decision-making test in the selection of elite male youth soccer players. |  | N/A |  | DT (reactive performance) |  | DT  stimulus response time ~  correct reactions |  | Inaccurate data reporting (age between 12-15 or 11-14?). |
| Taheri et al 2018 (86) | N/A |  | older woman  (n=35?, age: 61.1±2.8y, only females)  EXP1: biorhythm yoga (n=13)  EXP2: yoga (n=13)  CON: no exercise (n=13) |  | To investigate the effect of yoga based on biorhythm theory on the balance and selective attention in the older women. |  | no accurate information |  | COG (selective attention) |  | EXP 1, EXP 2: T1 > T2  CON: T1 ≂ T2 |  | Inaccurate data reporting (n=13 in each group but 35 in total?).  Effect sizes are not provided. |
| Teoldo et al 2022 (85) | Q2 |  | Brazilian soccer players  (n=48, age: 17.0±2.3y, only males) |  | To verify whether the peripheral perception and decision making of young soccer players are influenced by physical fatigue. |  | physical fatigue condition  and  control condition |  | PP (divided attention, peripheral detection) |  | non-significant differences in response to intervention |  | Lack of data on decision making within actual game settings. |
| Tsorbatzoudis et al 1998 (35) | N/A |  | overall age: 20±3.2y, only males  university students  (n=46)  EXP1: (n=12)  EXP2: (n=12)  CON1: (n=11)  CON2: (n=11)  top level cyclists  (n=12)  EXP3: (n=12) |  | To investigate the effect of physical exertion on cognitive functions of sport participants. |  | EXP1: high intensity exercise protocol (5min)  EXP2, EXP3: moderate intensity exercise protocol (30min)  CON1: performed the tests with a 10-min interval  CON2: performed the tests with a 35-min interval |  | DAUF (sustained attention) |  | non-significant differences between groups |  | Demographic data for subgroups are not provided.  Inaccurate data reporting (2 or 3 control groups?). |
| Tulppo et al 2014 (36) | Q1 |  | Finnish hockey players  EXP  (n=11, age: 25±6y, mass: 88±8kg, height: 1.8±0.06m, only males)  CON  (n=11, age: 24±4y, mass: 85±6kg, height: 1.8±0.07m, only males) |  | To evaluate the effects of transcranial bright light treatment via the ear canals on cognitive performance in professional ice hockey players during the competition season. |  | EXP: brain-targeted bright light treatment  CON: sham treatment (eliminated light production) |  | RT (decision time and motor time) |  | RT  motor time with a visual  warning signal  EXP: T1 > T2  CON: T1 ≂ T2 |  | Small sample size.  Lack of fMRI measurements to support the results with neuroimaging data.  Participants were aware of their group allocation. |
| Wilczyńska 2016 (67) | N/A |  | Polish rowers  (n=20, age: 23.9±2.6y) |  | To present possibilities of using computer tests in the assessment of chosen cognitive skills and an analysis of correlations between the mentioned variable and the level of optimism, self-efficacy, coping strategies, anxiety and perceived stress in Polish national team rowers. |  | N/A |  | RT (decision time and motor time)  DT (reactive performance) |  | RT (reaction time)  individual stimuli ~ simultaneously presented stimulus combinations  individual stimuli ~ sequentially presented stimulus combinations  DT ~ level of optimism, self-efficacy, different coping strategies, anxiety and perceived stress |  | Gender is not provided.  Seems to be an exploratory study. |
| Zheng et al 2024 (78) | Q1 |  | taekwondo athletes  (n=12, age: 20±3y, mass: 68.5±8kg, height: 1.76±0.06m, only males) |  | To investigate the impact of different levels of dehydration on cognitive function and the specific performance of kicking techniques among taekwondo athletes. |  | 70min taekwondo training bouts  5/week for 3w |  | RT (decision time and motor time)  COG (selective attention) |  | RT (reaction time)  hypohydrated group < severe hypohydration group |  | Gender is not provided.  Participants’ daily dietary habits and nutritional intake were not recorded. |
| Zisi et al 2009 (37) | N/A |  | rhythmic gymnasts  (n=33, age: 11.8±0.6y, only females) |  | To examine differences in selected cognitive, perceptual, motor abilities and psychological characteristics among elite rhythmic gymnasts. |  | N/A |  | RT (decision time and motor time)  DT (reactive performance) |  | non-significant differences between groups (different performance levels) |  | N/A |
| Zwierko 2006 (38) | N/A |  | soccer players  (n= 12, age: 22.3±2.7y)  PE students  (n= 12, age: 21.3±1.6y) |  | To examine the recognition and prediction of situations in the game of soccer. |  | N/A |  | ZBA (speed and movement in space) |  | ZBA  anticipation of the shots at  the goal (%)  soccer players > PE students |  | Gender is not provided.  Incorrect naming of the VTS test (ZBA is called motor-temporal test). |
| Zwierko 2007 (39) | N/A |  | handball players  (n=16, age: 21.9+1.1y)  nonathletes  (n=16, age: 20.1+1.8y) |  | To compare peripheral perception of handball players and nonathletes. |  | N/A |  | PP (divided attention, peripheral detection) |  | PP (omitted reactions)  handball athletes > nonathletes  PP (reaction time to visual stimuli)  handball athletes < nonathletes |  | Gender is not provided.  Post-hoc test results are not provided. |
| Zwierko et al 2008 (40) | N/A |  | Polish handball players  (n= 18, age: 21.2+3.1y, mass: 82.8+11.6kg, height: 1.85+0.06m, only males) |  | To examine changes in the level of peripheral perception induced by specific anaerobic effort in handball players and in relation to the running efficiency of the players. |  | running test to induce fatigue  (10x30m at maximum speed with 20s rest intervals) |  | PP (divided attention, peripheral detection) |  | PP  T1 < T2  visual angle/right, number of correct reactions (left & right)  T1 > T2  number of omitted reactions, median reaction time (left & right)  PP (number of correct reactions/right and number of omitted reactions) ~ times of the 10x30m runs |  | Post-hoc test results are not provided. |
| Zwierko et al 2010 (42) | Q4 |  | EXP: volleyball players  (n=12, age: 22.9±2.1y, only males)  CON: untrained students  (n=12, age: 21.9±1.5y) |  | To investigate the several aspects of reaction times to visual stimuli in volleyball players compared to non-athletic subjects. |  | N/A |  | RT (decision time and motor time)  [S1 - simple reaction time]  [S4 - choice reaction time]  PP (divided attention, peripheral detection) |  | volleyball players < non-athletes:  RT-S1 (pre-motor reaction time, total reaction time)  RT-S4 (pre-motor reaction time, total reaction time)  PP (total reaction time) |  | Gender for CON is not provided.  Incorrect naming of the VTS test (PP is called peripheral reaction time). |
| Zwierko et al 2014 (41) | N/A |  | physical education students  (n=22, age: 21.2±1.3y) |  | To investigate the effect of exercise on visuomotor processing. |  | effort test with incremental intensity on a cycloergometer |  | SIGNAL (selective attention) |  | non-significant differences in response to intervention |  | Gender for not provided.  Incorrect naming of the VTS test (SIGNAL is called Special Ability Signal test). |
| 2HAND: Two-Hand Coordination, 3D: Spatial Orientation, AMT: Adaptive Matrices Test, COG: Cognitrone Test, CON: control group, CORSI: Corsi-Block-Tapping-Test, DAUF: Continuous Attention Test, DT: Determination Test, EXP: experimental group, LVT: Visual Pursuit Test, MDT: Movement Detection Test, MLS: Motor Performance Series, PP: Peripheral Perception Test, RT: Reaction Test, SIGNAL: Signal Detection, SMK: Sensomotor Coordination, SPM: Raven’s Standard Progressive Matrices, STROOP: Stroop Interference Test, SWITCH: Task Switching, T: Time, TMT-L: Trail-Making Test, VISGED: Visual Memory Test, ZBA: Time/Movement Anticipation | | | | | | | | | | | | | |
