# Supplementary material for: Challenges in the practical application of the Vienna test system for assessing cognitive functions in the general, athletic and clinical populations: a global scoping review of experimental and observational studies
Source: Front Sports Act Living. 2026 Feb 23;8:1716584. doi: 10.3389/fspor.2026.1716584 (PMC12968311; doi:10.3389/fspor.2026.1716584)
Supplement: Supplementary file 4 [file Table3.docx]

| **Supplementary Table 3:** The application of Vienna Test System for assessing cognitive function in the clinical population | | | | | | | | | | | | | |
| --- | --- | --- | --- | --- | --- | --- | --- | --- | --- | --- | --- | --- | --- |
| **Study** | **SJR** |  | **Participants characteristics** |  | **Study objectives** |  | **Intervention** |  | **VTS cognitive tests** |  | **Key VTS-related findings** |  | **Remarks** |
| Chen et al 2016 (14) | Q1 |  | schizophrenia patients  (n=199, age: 44.0±9.9y, 77 females)  CON  (n=60, age: 41.1±9.6y, 26 females) |  | To examine the association between actigraphy-derived physical activity and cognitive performance adjusting for multiple covariates in patients with schizophrenia. |  | N/A |  | COG (selective attention) |  | COG  schizophrenia < CON |  | Cross-sectional design.  The study only  included two cognitive tests. |
| Dong et al 2023 (11) | Q1 |  | n = 464,  age: 34.1±10.8y, 18-62y, 189 females  ADHD patients  (n=227)  non-ADHD  (n=237) |  | To estimate base rates of noncredible performance in clinical evaluations of adult ADHD on embedded validity indicators (EVIs).  To examine the effect of the order of test administration on EVI failure rates, the association between cognitive underperformance and symptom overreporting, and the prediction of cognitive underperformance by clinical information. |  | N/A |  | **CFADHD package:**  TMT-L (visuomotor processing speed and cognitive flexibility)  WAF-S (selective attention)  NBV (working memory)  5POINT (figural fluency)  SWITCH (flexible task-switching ability)  WAF-V (perception and attention)  INHIB (response inhibition)  STROOP (interference color) |  | a total of 59.1% positive EVI throughout the entire battery  the most adequate and sensitive for detecting underperformance  WAF-S  WAF-V |  | Gender of subgroups is not reported.  VTS battery test order was not randomized. |
| Karakontaki  et al 2013 (89) | Q2 |  | COPD patients  (n=35, age: 59±7y, mass: 77±14kg, height: 1.7±0.07m, 9 females)  CON  (n=10, age: 55±5y, mass: 78±8kg, height: 1.7±0.05m, 2 females) |  | To investigate (a) whether subclinical neuropsychological deficits occur in stable COPD patients with mild hypoxemia, and (b) whether these deficits affect their driving performance. |  | N/A |  | RT (decision time and motor time)  COG (selective attention)  DT (reactive performance)  TAVTMB (PercPer in traffic) |  | RT, TAVTMB  COPD > CON |  | Incorrect naming of the VTS test (COG is called Selective Attention Test [SA] and DT is called Permanent Attention Test [PA]). |
| Kim et al 2009 (15) | Q4 |  | schizophrenia patients  (n=10, age: 28.7±1.7y,  5 females)  CON  (n=10, age: 25.7±9.6y,  5 females) |  | To determine which components are responsible for the slower RTs of patients with schizophrenia.  To explore the intra-individual variability of patients with schizophrenia in terms of their RTs for tasks. |  | N/A |  | RT (decision time and motor time) |  | RT (decision time)  schizophrenia > CON  inter-individual variability  intra-individual variability  schizophrenia > CON  performance:  schizophrenia: T1 ≂ T2  CON: T1 < T2 |  | Effect sizes are not provided. |
| Klasik et al 2011 (16) | Q3 |  | schizophrenia patients  (n=20) |  | To evaluate the effect of olanzapine treatment on selected cognitive functions in patients suffering from schizophrenia during an observation period of six-months. |  | 6-months of olanzapine treatment |  | COG (selective attention)  RT (decision time and motor time)  SIGNAL (selective attention) |  | COG (correct response)  T1 < T2 < T3 < T4 < T5  COG (incorrect response)  T1 > T2  T3 > T4  T2, T3, T4 < T5  RT (median reaction time)  T1 < T2 < T3 < T4 < T5  SIGNAL (correct and delayed responses)  T1 < T2, T3, T4, T5  T2 < T4, T5  T3 < T5 |  | Demographic data and gender are not reported.  No control group.  Effect sizes are not provided. |
| Klasik et al 2012 (12) | Q3 |  | depression patients  GROUP 1  (n=20)  GROUP 2  (n=20)  GROUP 3  (n=20) |  | To analyze short-term memory and attention deficits in patients suffering from recurrent depressive disorder treated with psycho- therapy and pharmacotherapy. |  | 8-week long interventions  GROUP 1: psychodynamic psychotherapy  GROUP 2: pharmacotherapy and psychotherapy  GROUP 3:  medication with sertraline |  | COG (selective attention)  SIGNAL (selective attention) |  | CORSI (1^st^ examination)  GROUP 1 > GROUP 3  CORSI (2^nd^ examination)  GROUP 1 < GROUP 2  GROUP 1 < GROUP 3  GROUP 3 < GROUP 2  SIGNAL (1^st^ examination)  GROUP 1 > GROUP 2  GROUP 1 > GROUP 3  GROUP 3 > GROUP 2  SIGNAL (2^nd^ examination)  GROUP 1 < GROUP 2  GROUP 1 < GROUP 3  GROUP 3 < GROUP 2 |  | Demographic data and gender are not reported.  No healthy control group.  The interventions are not described in details.  Effect sizes are not provided.  Results reporting for CORSI is incorrect, tables are correct. |
| Kloek et al 2020 (93) | Q1 |  | EXP: pneumococcal meningitis patients  (n=80, age: med: 63y, IQR: 56-69y, 41 females)  CON: partners or proxies of patients  (n=69, age: med: 65y, IQR: 54-68y, 34 females) |  | To perform a cross-sectional cohort study on long-term neurologic, cognitive and quality-of- life outcome in adults surviving pneumococcal meningitis. |  | N/A |  | **COGBAT package:**  TMT-L (visuomotor processing speed and cognitive flexibility)  WAF-A (reaction and attention intensity)  NBV (working memory)  FGT (learning ability, short- and long-term memory)  TOL-F (planning ability)  INHIB (response inhibition) |  | differences between  EXP and CON:  WAFA  TMT-L  overall test score |  | The study has a selection bias.  Patients who could not be reliably assessed with the neuropsychologic test battery as a result of mental or physical limitations were excluded.  Not a randomized controlled trial. |
| Miah et al 2012 (13) | Q1 |  | EXP 1: de novo PD patients  (n=23, age: 59.5±7.2y,  8 females)  EXP 2: mild to moderately advanced, medicated PD patients  (n=55, age: 63.9±6.3y,  27 females)  CON: healthy controls  (n=21, age: 60.3±6.5y,  10 females) |  | To establish the cognitive profile of newly diagnosed untreated (de novo) patients with Parkinson’s disease (PD) and more advanced, treated patients, and to determine the effects of dopamine replacement therapy. |  | dopamine replacement therapy |  | PERSEV |  | relative redundancy of the second order  (deficit to generate random motor sequences)  EXP 2 < CON |  | Incorrect naming of the VTS test (PERSEV is called VPT).  Cross-sectional design.  The cognitive effects should be cautiously interpreted. |
| Ramm et al 2017 (90) | Q1 |  | oEDS: patients with objective excessive daytime sleepiness  (n=23, age: 31.8±12.8y, 16 females)  sEDS: patients with subjective excessive daytime sleepiness  (n=13, age: 32.2±14.6y, 11 females)  CON: healthy controls  (n=20, age: 32.6±11.3y, 13 females) |  | To evaluate an attention test as a discriminative tool to measure neurocognitive impairment in patients with disorders of hypersomnolence. |  | N/A |  | WAF-A (reaction and attention intensity)  WAF-G (parallel processing and selectivity of attention)  WAF-S (selective attention)  WAF-V (perception and attention) |  | mean reaction time  1) WAF-A (tonic alertness)  oEDS > CON  WAF-G (divided attention)  oEDS > CON  WAF-S (selective attention)  oEDS > CON  sEDS > CON  WAF-V (vigilance/sustained attention)  oEDS > CON  sEDS > CON |  | Comparison between different central disorders of hyper-somnolence should be interpreted with caution as subgroups consisted of only few patients. |
| Ramm et al 2019 (88) | Q1 |  | NT1: patients with narcolepsy type 1  (n=10, age: 26.7y,  20-33.4y,  3 females)  IH: patients with idiopathic  hypersomnia  (n=14, age: 33.6y,  26.8-40.4y,  11 females)  sEDS: patients with subjective EDS  (n=14, age: 31.4y,  23.1-39.7y,  12 females)  CON: healthy controls  (n=20, age: 32.6y,  27.3-37.9y,  13 females) |  | To explore attentional profiles in disorders of chronic excessive daytime sleepiness (EDS). |  | N/A |  | **SLEEP package:**  WAF-V (perception and attention)  WAF-S (selective attention) |  | WAF-V  1) mean reaction time  GROUP main effect (post-hoc tests are not shown)  2) omissions and errors  1^st^ half  NT1 > sEDS  2^nd^ half  NT1, IH, sEDS > CON  WAF-S  1) mean and SD of reaction time  GROUP main effect (post-hoc tests are not shown)  2) omissions and errors (compared to CON)  sEDS: T1 < T2 |  | It is an exploratory study.  Relatively small sample size.  High interindividual variation of the attentional performances. |
| Sabatowski et al 2014 (9) | N/A |  | patients who had completed previous tapentadol PR trials for severe low back or osteoarthritis pain  (n=38, age: 58.0±7y,  24 females) |  | To evaluate the effects of tapentadol prolonged release on driving ability. |  | after at least 6 weeks of dose stability, patients continued taking tapentadol PR (50–250 mg twice daily) and could take supplemental immediate-release tapentadol 50 mg, except on the day before or day of the driving test |  | **TRAFFIC PLUS package:**  COG (selective attention)  WRB-TV (subjectively accepted risk level)  ATAVT (visual orientation, perceptual speed)  VIGIL (sustained attention)  DT (reactive performance)  LVT (visual orientation, attention)  PP (divided attention, peripheral detection)  2HAND (visual-motor coordination)  RT (decision time and motor time)  AMT (intelligence) |  | successful outcome:  > 70% of patients  COG, ATAVT, DT, PP, WRB-TV, 2HAND, VIGIL, LVT  50% < patients < 70%  RT  31.4% of patients  AMT |  | It is an exploratory study.  No pre-study measurements of driving ability were performed.  Only short-term effects were evaluated. |
| Schmidt et al 2023 (92) | Q2 |  | CP: patients with chronic pain  (n=40, age: 53.3±13.8y,  25 females)  CON: healthy controls  (n=41, age: 46.7±18.4y,  26 females) |  | To conduct a comprehensive assessment of self-rated and objectively assessed cognitive differences between patients with chronic pain and healthy controls. |  | N/A |  | **COGBAT package:**  TMT-L (visuomotor processing speed and cognitive flexibility)  WAF-G (parallel processing and selectivity of attention)  COG (selective attention)  ATAVT (visual orientation, perceptual speed)  DT (reactive performance)  RT (decision time and motor time)  LVT (visual orientation, attention)  NBV (working memory)  FGT (learning ability, short- and long-term memory)  TOL-F (planning ability)  INHIB (response inhibition) |  | significant differences in each COGBAT test |  | The authors did not consider the current pain intensity. |
| Shmygalev et al 2011 (91) | Q1 |  | EXP: buprenorphine patients  (n=30, age: 36.6±6.3y,  2 females)  CON: healthy controls  (n=90, age: 37.1±6.0y,  6 females) |  | To use tests that predict driving ability to assess the impact of long-term substitution therapy with sublingual buprenorphine in patients suffering from opioid dependency. |  | N/A |  | COG (selective attention)  DT (reactive performance)  ATAVT (visual orientation, perceptual speed)  2HAND (visual-motor coordination)  VIGIL (sustained attention) |  | 1) DT  right reactions EXP > CON  mean reaction time EXP < CON  2) COG  right and wrong answers  EXP > CON  3) 2HAND  average time, time of track, score  EXP < CON  4) VIGIL  right answers  EXP > CON  wrong answers  EXP < CON |  | Incorrect naming of the VTS test (ATAVT is called TAVT and VIGIL is called VIG).  Not a well-controlled study in terms of patient recruitment strategy. |
| Stubbs et al 2017 (17) | Q1 |  | EXP: schizophrenia patients  (n=199, age: 44.0±9.9y,  77 females)  CON: healthy controls  (n=60, age- and gender-matched) |  | To investigate sedentary behavior levels in people with schizophrenia compared to controls. |  | Sedentary behavior and physical activity (PA) were captured for 7 consecutive days with an accelerometer. |  | COG (selective attention)  RT (decision time and motor time) |  | COG, RT  EXP < CON |  | Cross-sectional design.  The study included people with established psychosis (generalizability). |
| 2HAND: Two-Hand Coordination, 5POINT: 5-Point Test, ADHD: Attention-Deficit/Hyperactivity Disorder, AMT: Adaptive Matrices Test, ATAVT: Adaptive Tachistoscopic Traffic Perception Test, CFADHD: Cognitive Functions ADHD Test set, COG: Cognitrone Test, COGBAT: Cognitive Basic Assessment Test set, CON: control group, COPD: Chronic Obstructive Pulmonary Disease, DT: Determination Test, EXP: experimental group, FGT: Figural Memory Test, INHIB: Response Inhibition, IQR: interquartile range, LVT: Visual Pursuit Test, NBV: N-Back Verbal, PD: Parkinson’s disease, PP: Peripheral Perception Test, SIGNAL: Signal Detection, SWITCH: Task Switching, TAVTMB: Tachistoscopic Traffic Test, TMT-L: Trail-Making Test, TOL-F: Tower of London Test - Freiburg Version, VIGIL: Vigilance Test, WAF: Perception and Attention Functions Battery, WRB-TV: Vienna Risk-Taking Test Traffic | | | | | | | | | | | | | |
